# Effects of exercise based on ACSM recommendations on bone mineral density in individuals with osteoporosis: a systematic review and meta-analyses of randomized controlled trials

**DOI:** 10.3389/fphys.2023.1181327

**Published:** 2023-07-17

**Authors:** Wenlai Cui, Dong Li, Yueshuai Jiang, Yang Gao

**Affiliations:** ^1^ School of Dance and Martial Arts, Capital University of Physical Education and Sports, Beijing, China; ^2^ Department of International Cultural Exchange, Chodang University, Muan County, Republic of Korea

**Keywords:** osteoporosis, bone mineral density, ACSM recommendations, exercise intervention, exercise dose

## Abstract

**Purpose:** To analyze the effects of different exercise dose on lumbar spine and femoral neck bone mineral density (BMD) in individuals with osteoporosis (OP).

**Design:** A systematic search was conducted in four electronic databases, namely, PubMed, Embase, Web of Science, and Cochrane, with the topic of the impact of exercise on BMD in individuals with OP. Randomized controlled trials comparing exercise intervention with no intervention were identified, and changes in lumbar spine and femoral neck BMD were reported and evaluated using standardized mean difference (SMD) and 95% confidence interval (95% CI). The intervention measures in the studies were evaluated and categorized as high adherence with the exercise testing and prescription recommendations for individuals with OP developed by the American College of Sports Medicine (ACSM) or low/uncertainty adherence with ACSM recommendations. A random effects model was used to conduct meta-analyses and compare the results between subgroups.

**Results:** A total of 32 studies involving 2005 participants were included in the analyses, with 14 studies categorized as high adherence with ACSM recommendations and 18 studies categorized as low or uncertain adherence. In the analyses of lumbar spine BMD, 27 studies with 1,539 participants were included. The combined SMD for the high adherence group was 0.31, while the combined SMD for the low or uncertain adherence group was 0.04. In the analyses of femoral neck BMD, 23 studies with 1,606 participants were included. The combined SMD for the high adherence group was 0.45, while the combined SMD for the low or uncertain adherence group was 0.28. Within resistance exercise, the subgroup with high ACSM adherence had a greater impact on lumbar spine BMD compared to the subgroup with low or uncertain ACSM adherence (SMD: 0.08 > −0.04). Similarly, for femoral neck BMD, resistance exercise with high ACSM adherence had a higher SMD compared to exercise with low or uncertain ACSM adherence (SMD: 0.49 > 0.13).

**Conclusion:** The results suggest that exercise interventions with high adherence to ACSM recommendations are more effective in improving lumbar spine and femoral neck BMD in individuals with OP compared to interventions with low or uncertain adherence to ACSM recommendations.

**Systematic Review Registration**: PROSPERO, identifier CRD42023427009

## 1 Introduction

Osteoporosis (OP) is a skeletal disease characterized by a decrease in bone mineral density (BMD), changes in bone tissue microstructure, and an increased risk of fractures (Consensus development conference: Diagnosis, prophylaxis, and treatment of osteoporosis, 1993; Sugerman, 2014; LeBoff et al., 2022). OP can be classified into primary and secondary types (Bonura, 2009), with primary OP being common in postmenopausal women and older adults (Wei et al., 2015). Although OP does not directly cause death, it increases the risk of falls and fractures (Bonura, 2009; Yu et al., 2019). Fractures usually occur in the vertebrae (lumbar spine), proximal femur (hip), and distal forearm (wrist) (Hadji et al., 2013; Sugerman, 2014; LeBoff et al., 2022), severely affecting the health and quality of life of individuals. In 2010, it was estimated that 22 million women and 5.5 million men in the European Union had OP (Svedbom et al., 2013). In the United States, OP affects more than 25 million people (Consensus development conference: Diagnosis, prophylaxis, and treatment of osteoporosis, 1993; Bonura, 2009). A study in China found that the incidence of OP in the population over 50 years old was 6.46% for men and 29.13% for women (Zeng et al., 2019). Due to changes in the population structure, the number of individuals with OP is gradually increasing, causing a huge burden on society and patient families (Burge et al., 2007). Currently, OP treatment methods include drug therapy (chemical synthetic drugs that inhibit bone resorption and promote bone formation, such as bisphosphonates, parathyroid hormone analogs, denosumab, etc.) and non-pharmacological interventions (exercise, avoidance of smoking and excessive alcohol consumption, etc.) (LeBoff et al., 2022), but special drug treatments usually have certain side effects (Skjødt et al., 2019), indirectly leading to a decrease in quality of life. Exercise, as an important non-pharmacological intervention, can not only improve OP symptoms (Tong et al., 2019) but also increase the happiness index of residents.

Research has shown that after exercise intervention, individuals with OP demonstrate significant improvements in quality of life, pain scores, and balance performance (Marcu et al., 2015; FILIPOVIĆ et al., 2021), but there is still controversy regarding changes in BMD (Wei et al., 2015). BMD is an important indicator for diagnosing individuals with OP (Sugerman, 2014), and increasing BMD through exercise is crucial for preventing and treating OP (Gregson et al., 2022). A meta-analyses has investigated the effects of different exercise interventions on BMD in postmenopausal women or individuals with bone loss, and the results show that exercise can improve BMD (Kemmler et al., 2020). Mind-body exercise is the most effective type of exercise for improving BMD, followed by resistance exercise, which is more effective than aerobic exercise in improving BMD (Zhang et al., 2022). M. Shojaa et al.’s meta-analyses studied the effects of dynamic resistance training on BMD in postmenopausal women and emphasized exercise parameters. They found that lower training frequencies (<2 sessions/week) resulted in better BMD changes than higher training frequencies (≥2 sessions/week) (Shojaa et al., 2020). A recent systematic review analyzed the effects of different exercise intensities on BMD in postmenopausal women with OP and reported that high-intensity resistance training was better than moderate-intensity resistance training for improving lumbar spine BMD (Kitagawa et al., 2022). Currently, exercise has been proven to have a beneficial effect on individuals with OP as a non-pharmacological treatment method (Ernst, 1998; Cheung and Giangregorio, 2012), but because we still do not fully understand the mechanisms by which exercise prevents and treats OP (Tong et al., 2019), a large amount of experimental data is still needed to determine the optimal exercise dose for preventing and treating OP.

The American College of Sports Medicine (ACSM) has developed recommended exercise prescriptions for apparently healthy adults, including detailed descriptions of the recommended dose of cardiorespiratory exercise, resistance exercise, and flexibility exercise for individuals with OP (Garber et al., 2011; Moseng et al., 2017). However, it is currently unclear whether exercise interventions based on the ACSM recommendations will have a greater impact on BMD in individuals with OP than exercise interventions with low or uncertain adherence. The purpose of this systematic review is to compare the effects of high adherence and low or uncertain adherence exercise interventions based on the ACSM recommendations on BMD in individuals with OP.

## 2 Materials and methods

The systematic review and meta-analyses will be reported based on the Preferred Reporting Items for Systematic Reviews and Meta-Analyses (PRISMA) statement and registered in PROSPERO (CRD42023427009).

### 2.1 Search strategy

We searched PubMed, Embase, Web of Science, and Cochrane databases from their inception to 5 January 2023, using a search strategy based on the PICOS principle, focusing on the study population, intervention, and research methodology. The search terms included the following: (“Osteoporosis” or “Osteoporoses” or “osteopenic” or “osteoporotic” or “osteopenia” or “Post-Traumatic Osteoporoses” or “Senile Osteoporoses” or “Bone Loss” or “Bone Losses” or “Bone density” or “Bone mineral density” or “Low bone mass”) AND (“Exercise” or “Exercises” or “Physical Activity” or “physical exercise” or “Training” or “Trainings” or “Motor Activity” or “Tai Chi” or “Vibration” or “yoga” or “wuqinxi” or “baduanjin” or “yijinjing” or “kickboxing” or “Pilates” or “Balance” or “Resistance”) AND (“Randomized controlled trial” or “controlled clinical trial” or “randomized” or “placebo” or “randomly”). The detailed search strategy is shown in Supplementary Appendix SA1. We also conducted a manual search for the bibliographies of relevant reviews and included studies. When necessary, we contacted the study authors to obtain additional information.

### 2.2 Criteria for selection of studies

The studies we included met the following criteria: (a) published randomized controlled trials (RCTs); (b) study subjects were OP or low bone mass population; (c) intervention could be any land-based exercise program, including resistance training, aerobic exercise, flexibility exercise, etc.; (d) control intervention could be no treatment or any treatment unrelated to exercise, thus studies comparing different exercise interventions were excluded; (e) reporting of BMD at the lumbar spine or femur neck in the study results; (f) BMD was determined by dual-energy X-ray absorptiometry (DXA) or dual-photon absorptiometry (DPA).

We excluded studies that included the following: (a) reports, conference proceedings, reviews, etc. were not considered; (b) studies based on aquatic exercise and those without a comparison between a land-based exercise intervention and a non-exercise group were excluded; (c) populations with related bone metabolic diseases; (d) studies that simultaneously received special drug treatments during the exercise intervention were excluded; (e) duplicate experimental data from multiple publications from the same study were excluded.

Two authors (WLC and DL) independently screened titles and abstracts of the literature that met the inclusion criteria. If one of the authors considered a study to meet the criteria, the full text of the article was obtained. Then, two authors independently assessed whether the full text met the requirements. If there was no agreement, the third author (YG) made a decision, and consensus was reached through discussion. There were no restrictions on subject age, gender, body mass index, publication date, or language.

### 2.3 Data synthesis and analyses

Data extraction was performed independently by two authors (WLC and YSJ) for the included studies. BMD of the lumbar spine and femoral neck were considered as the primary outcomes of this study. An Excel spreadsheet was pre-designed to extract relevant data, including publication characteristics (title, author names, country, year of publication), methodological features (number of study groups, design of each group, intervention measures, sample size), participant characteristics (age, gender ratio, BMI), exercise features (intervention frequency, exercise intensity, exercise time, repetition, set), risk assessment, and outcome features.

When extracting outcome data, if the study did not clearly state the post-intervention results data but presented them in graphical form, Engauge Digitizer software was used to extract the data. For studies with multiple follow-up evaluations, we only extracted the data immediately after the intervention.

After data extraction, the exercise intervention was evaluated for dose and adherence. The exercise intervention dose included in the study were evaluated based on the ACSM recommendations for the development and maintenance of cardiorespiratory, muscular, skeletal, and neural function in individuals with OP (Garber et al., 2011). Two authors (WLC and YG) independently assessed the exercise interventions for each study according to different criteria defined by the ACSM recommendations for each aspect of exercise dose (including frequency, intensity, duration, etc.), in order to assess adherence with exercise dose (Table 1).

**TABLE 1:** The ACSM recommendations for cardiorespiratory fitness, muscular strength and flexibility in apparently healthy adults.

Exercise dose	Cardiorespiratory exercise	Resistance exercise	Flexibility exercise
Frequency	4–5 days/week	1–2 days/week (non-consecutive days), gradually increasing to 2–3 days/week.	5–7 days/week
Intensity/workload	Moderate intensity, 40%–59% VO^2^R/HRR, CR-10 scale rating of 3–4	Adjust the resistance, with the last two sets being challenging. High intensity training can be performed if tolerable.	Stretch until you feel your muscles being pulled tight or a slight discomfort.
Duration	Gradually increase from 20 min to at least 30 min (up to 45–60 min)	Starting with one set of 8–12 repetitions, increase to two sets after about 2 weeks. Perform no more than 8–10 exercises per session.	Static stretching held for 10–30 s, repeated 2–4 times.

HRR, Heart rate reserve. VO^2^R, oxygen uptake reserve.

The scoring range for each exercise indicator was 0–2 points. 2 points: met the criteria; 1 point: uncertain; 0 points: definitely did not meet the criteria. If the two authors did not come to a similar conclusion, they discussed with a third author to reach a consensus. Based on this scoring system, we calculated the proportion of exercise dose that met the recommended dose of exercise by ACSM for each study. When the proportion was ≥ 70%, we classified it as high adherence with ACSM recommendations; when the proportion was <70%, we classified it as low adherence or uncertain adherence with ACSM recommendations.

### 2.4 Statistical analyses

Meta-analyses were performed using STATA 16.0 to compare the results of the included studies. The studies were divided into two groups in the meta-analyses, representing high adherence and low or uncertain adherence with the ACSM recommendations. The heterogeneity among studies within each subgroup was evaluated using the Higgins I^2^ statistic and interpreted according to the Cochrane Handbook’s recommendations (Deeks et al., 2019): The level of heterogeneity was categorized as small (I^2^ ≤ 25%), moderate (25% < I^2^ ≤ 50%), substantial (50% < I^2^ ≤ 75%), or considerable (I^2^ > 75%). In the heterogeneity test, if I^2^ ≤ 50%, a fixed-effect model was used to test the effect size; if I^2^>50%, a random-effects model was used, and the effect size was represented by the standardized mean difference (SMD) with a 95% confidence interval (95% CI). When heterogeneity is high, we further conducted meta-regression analyses to explore the sources of heterogeneity by examining the potential research characteristics that may contribute to it. The likelihood of publication bias was evaluated by constructing funnel plots of the effect size relative to the standard error for each study and testing for asymmetry using Begg’s rank correlation test and Egger’s linear regression test, with *p* < 0.05 indicating statistical significance. Sensitivity analyses was also performed to examine the robustness of the study results by excluding each study one by one.

### 2.5 Quality appraisal

The methodological quality of the included studies was assessed by two pairs of authors (LCW and YG, DL and YSJ) using the quality assessment criteria recommended by the Cochrane Collaboration (Higgins et al., 2011). All studies included in this review were randomized controlled trials. According to the Cochrane Handbook, when including randomized controlled trials, the recommended tool is the revised version of the Cochrane tool, called the Risk of Bias tool (Rob 2) (Sterne et al., 2019). The Rob 2 tool provides a framework for assessing the risk of bias of individual outcomes in any type of randomized trial. The evaluation indicators include random sequence generation, allocation concealment, blinding of participants and personnel, blinding of outcome assessment, incomplete outcome data, selective reporting, and other biases, and reviewers rate different studies based on the Cochrane Handbook. The bias risk of each area is divided into three levels: “low risk,” “some concerns,” and “high risk.” If all areas are evaluated as “low risk,” then the overall bias risk is “low.” If some areas are evaluated as “some concerns” and no areas are evaluated as “high risk,” then the overall bias risk is “some concerns.” If one area is evaluated as “high risk,” then the overall bias risk is “high” (Cumpston et al., 2019).

## 3 Results

### 3.1 Study selection

**FIGURE 1:**
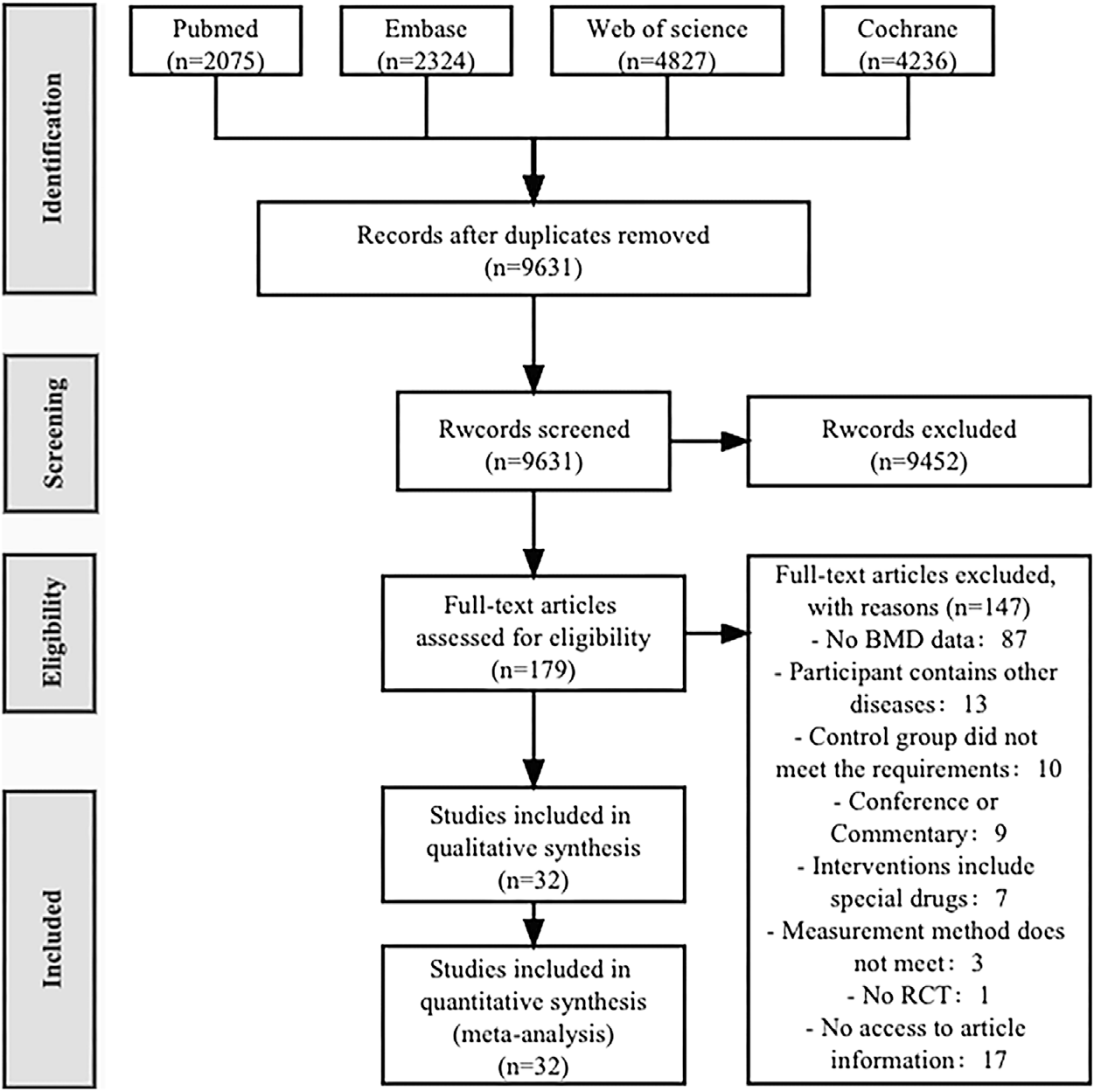
PRISMA Study flow diagram.

A total of 13,462 articles were retrieved from four databases (PubMed 2,075, Embase 2,324, Web of Science 4,827, Cochrane 4,236). After removing duplicates (2,473), 9,631 articles were left. After reviewing the titles and abstracts, 179 articles were selected for full-text reading, and finally, 32 articles were included in this review (Nelson et al., 1994; Bravo et al., 1996; Hartard et al., 1996; Iwamoto et al., 1998; Iwamoto et al., 2001; Chien et al., 2000; Liu-Ambrose et al., 2004; Korpelainen et al., 2006; Bergström et al., 2008; Brentano et al., 2008; Hourigan et al., 2008; Bocalini et al., 2010; Bolton et al., 2012; Marchese et al., 2012; Wayne et al., 2012; Basat et al., 2013; Mosti et al., 2013; Hakestad et al., 2015; Liu et al., 2015; Wang et al., 2015; Watson et al., 2015; 2018; Shen et al., 2018; Posch et al., 2019; ElDeeb and Abdel-Aziem, 2020; Hettchen et al., 2021; Kistler-Fischbacher et al., 2021; Rajapakse et al., 2021; Cheng et al., 2022; Kienberger et al., 2022; Lin et al., 2022; Waltman et al., 2022) (Figure 1).

### 3.2 Study characteristics

A total of 32 studies including 2005 participants were included, with 1,005 participants in the intervention group and 1,000 participants in the control group. The age range of the participants was 50–83 years, and 22 studies reported participants' BMI, which ranged from 18.4 Kg/m^2^ to 29.82 Kg/m^2^ (Nelson et al., 1994; Bravo et al., 1996; Iwamoto et al., 1998; 2001; Korpelainen et al., 2006; Bergström et al., 2008; Bocalini et al., 2010; Bolton et al., 2012; Wayne et al., 2012; Basat et al., 2013; Hakestad et al., 2015; Watson et al., 2015; Watson et al., 2018; Posch et al., 2019; ElDeeb and Abdel-Aziem, 2020; Hettchen et al., 2021; Kistler-Fischbacher et al., 2021; Rajapakse et al., 2021; Cheng et al., 2022; Kienberger et al., 2022; Lin et al., 2022; Waltman et al., 2022). Only three studies involved male participants (Shen et al., 2018; Cheng et al., 2022; Lin et al., 2022), comprising approximately 3.3% of the total number of participants. Six studies involved two or more exercise groups (Liu-Ambrose et al., 2004; Brentano et al., 2008; Basat et al., 2013; Liu et al., 2015; Kistler-Fischbacher et al., 2021; Cheng et al., 2022). One study described the study population as postmenopausal women with OP without specifying the age range (Brentano et al., 2008), while another study involved postmenopausal women with forearm fractures (Bergström et al., 2008). Among these studies, six were conducted in China, five in Australia, four in the United States, and two each in Germany, Brazil, Japan, and Austria, while Turkey, Sweden, Norway, Canada, Italy, Egypt, Finland, and Colombia each had one study (Table 2). Participants were mainly recruited through hospital orthopedic clinics, community recruitment, and advertising media.

**TABLE 2:** Study characteristics.

Author, year	Country	Sample size (n)		Age (years)		BMI (Kg/m^2^)	
		IG (F:M)	CG (F:M)	IG	CG	IG	CG
Shen et al. (2018)	China	22:18	26:14	75.5 (2.6)	74.3 (4.3)	NR	NR
Basat et al. (2013)	Turkey	11:0	12:0	55.9 (4.9)	56.2 (4.0)	25.0 (4.7)	27.5 (3.7)
Lin et al. (2022)	Taiwan, China	29:12	27:7	50–85	50–85	24.13 (3.30)	24.06 (2.67)
Liu et al. (2015)	China	48:0	42:0	63.23 (7.56)	61.87 (8.29)	NR	NR
Hartard et al. (1996)	Germany	16:0	15:0	63.6 (6.2)	67.4 (9.7)	NR	NR
Hourigan et al. (2008)	Australia	50:0	48:0	61.5 (8.2)	61.9 (9.6)	NR	NR
Brentano et al. (2008)	Brazil	9:0	9:0	NR	NR	NR	NR
Bergström et al. (2008)	Sweden	48:0	44:0	58.9 (4.3)	59.6 (3.6)	24.4 (2.6)	24.9 (2.3)
Bocalini et al. (2010)	Brazil	13:0	12:0	66 (9)	64 (8)	28 (1.3)	29 (2.2)
Mosti et al. (2013)	Norway	8:0	8:0	61.9 (5.0)	66.7 (7.4)	NR	NR
Wayne et al. (2012)	United States	43:0	43:0	58.8 (5.6)	60.4 (5.3)	25.8 (4.2)	24.5 (4.0)
Bravo et al. (1996)	Canada	61:0	63:0	59.6 (5.82)	59.9 (6.36)	24.3 (4.05)	24.3 (3.71)
Watson et al. (2018)	Australia	43:0	43:0	65 (5)	65 (5)	24.5 (4.6)	23.7 (3.2)
Watson et al. (2015)	Australia	12:0	16:0	65.3 (3.9)	66.7 (5.4)	23.2 (3.4)	23.8 (3.9)
Chien et al. (2000)	Taiwan, China	22:0	21:0	57.1 (8.6)	57.0 (5.4)	NR	NR
Kienberger et al. (2022)	Austria	19:0	19:0	62.8 (6.8)	58.7 (8.2)	25.5 (4.0)	24.0 (3.7)
Cheng et al. (2022)	China	12:7	12:8	64.82 (10.05)	65.68 (10.50)	23.10 (4.50)	22.85 (4.35)
Nelson et al. (1994)	United States	20:0	19:0	61.1 (3.7)	57.3 (6.3)	24.4 (2.5)	23.1 (2.2)
Bolton et al. (2012)	Australia	19:0	18:0	60.3 (5.6)	56.3 (4.7)	25.2 (4.3)	25.0 (4.4)
Marchese et al. (2012)	Italy	11:0	11:0	64.09 (8.90)	59.73 (1.19)	NR	NR
Posch et al. (2019)	Austria	20:0	20:0	69.6 (5.3)	67.4 (6.8)	25.1 (3.2)	24.4 (3.7)
ElDeeb and Abdel-Aziem (2020)	Egypt	22:0	21:0	55.09 (4.19)	57.29 (4.44)	28.36 (1.31)	28.28 (1.54)
Rajapakse et al. (2021)	United States	25:0	29:0	55.2–63.4	54.5–62.1	26.2 (3.62)	25.3 (3.55)
Iwamoto et al. (1998)	Japan	15:0	20:0	64.8 (6.1)	64.8 (5.7)	20.1 (2.0)	19.9 (2.1)
Korpelainen et al. (2006)	Finland	84:0	76:0	72.9 (1.1)	72.8 (1.2)	25.7 (3.4)	25.5 (3.5)
Hakestad et al. (2015)	Norway	42:0	38:0	65.5 (7.1)	63.9 (7.1)	24.2 (4.1)	24.3 (2.8)
Iwamoto et al. (2001)	Japan	8:0	20:0	65.3 (4.7)	64.9 (5.7)	19.7 (1.3)	20.5 (2.6)
Hettchen et al. (2021)	Germany	27:0	27:0	53.6 (2.0)	54.5 (1.6)	23.7 (3.4)	24.9 (4.8)
Liu-Ambrose et al. (2004)	Colombia	33:0	32:0	78.9 (2.8)	79.5 (3.2)	NR	NR
Waltman et al. (2022)	United States	91:0	93:0	54.5 (3.0)	54.3 (3.3)	25.2 (4.3)	25.9 (5.2)
Kistler-Fischbacher et al. (2021)	Australia	48:0	52:0	63.3 (6.4)	63.6 (4.9)	26.2 (4.6)	25.5 (4.5)
Wang et al. (2015)	China	37:0	35:0	57.93 (3.22)	58.54 (3.37)	NR	NR

Numbers are mean (SD) unless otherwise stated. NR, Not Reported; IG, Intervention group; CG, Control group; F:M, Female: Male.

The main intervention durations ranged from 12 weeks to 30 months, and the exercise frequency ranged from 2 to 7 days per week. All studies included exercise interventions that involved supervised exercise or home-based exercise, and the types of interventions mainly included resistance exercise, balance training, vibration exercise, Tai Chi, and Baduanjin (Table 3). After categorizing the interventions, 20 studies involved the exercise dose of cardiorespiratory exercise, 24 studies involved the exercise dose of resistance exercise, and 8 studies involved the exercise dose of flexibility exercise (Table 4).

**TABLE 3:** Characteristics of the study intervention.

Study	Interventions	Outcome LS (Mean ± SD)		Outcome FN (Mean ± SD)		Length of intervention
		IG	CG	IG	CG	
Shen et al. (2018)	Yi Jin Jing	NR	NR	0.790 **±** 0.130	0.680 **±** 0.110	6 Months
Basat et al. (2013)	Strengthening exercise	0.925 **±** 0.039	0.911 **±** 0.048	0.842 **±** 0.083	0.840 **±** 0.069	6 Months
Lin et al. (2022)	Kickboxing exercise	NR	NR	0.590 **±** 0.100	0.620 **±** 0.100	12 Weeks
Liu et al. (2015)	Eight-Section Brocade	0.649 **±** 0.035	0.638 **±** 0.032	0.547 **±** 0.018	0.532 **±** 0.035	12 Months
Hartard et al. (1996)	Strength Training	0.874 **±** 0.210	0.822 **±** 0.125	0.736 **±** 0.137	0.670 **±** 0.080	6 Months
Hourigan et al. (2008)	Balance and weight-bearing exercise program	1.070 **±** 0.190	1.110 **±** 0.160	0.870 **±** 0.100	0.900 **±** 0.130	20 Weeks
Brentano et al. (2008)	Strength training	0.935 **±** 0.086	0.960 **±** 0.134	0.700 **±** 0.060	0.700 **±** 0.080	24 Weeks
Bergström et al. (2008)	Physical training	0.957 **±** 0.077	1.014 **±** 0.108	NR	NR	12 Months
Bocalini et al. (2010)	Moderate Resistive Training	0.882 **±** 0.004	0.875 **±** 0.008	0.701 **±** 0.004	0.693 **±** 0.005	24 Weeks
Mosti et al. (2013)	Maximal strength training	0.762 **±** 0.067	0.818 **±** 0.121	0.655 **±** 0.088	0.635 **±** 0.059	12 Weeks
Wayne et al. (2012)	Tai Chi exercise	NR	NR	0.681 **±** 0.063	0.685 **±** 0.069	9 Months
Bravo et al. (1996)	Weight-bearing exercises	0.916 **±** 0.154	0.920 **±** 0.180	0.747 **±** 0.100	0.745 **±** 0.112	12 Months
Watson et al. (2018)	High-intensity resistance and impact training	0.846 **±** 0.116	0.807 **±** 0.098	0.700 **±** 0.084	0.670 **±** 0.059	8 Months
Watson et al. (2015)	High-intensity progressive resistance training	0.864 **±** 0.124	0.771 **±** 0.126	0.708 **±** 0.112	0.667 **±** 0.075	8 Months
Chien et al. (2000)	Aerobic Exercise	0.760 **±** 0.106	0.753 **±** 0.091	0.707 **±** 0.081	0.648 **±** 0.044	6 Months
Kienberger et al. (2022)	Resistance training	−1.810 **±** 0.620	−1.830 **±** 0.665	NR	NR	12 Months
Cheng et al. (2022)	Walking	1.060 **±** 0.090	0.880 **±** 0.050	NR	NR	24 Weeks
Nelson et al. (1994)	High-intensity strength training	1.029 **±** 0.220	0.947 **±** 0.203	0.858 **±** 0.176	0.806 **±** 0.142	1 Year
Bolton et al. (2012)	Exercises for hip strength, muscle strength and balance	0.893 **±** 0.097	0.897 **±** 0.128	NR	NR	52 Weeks
Marchese et al. (2012)	Weight-bearing exercise	1.070 **±** 0.120	1.040 **±** 0.120	NR	NR	24 Weeks
Posch et al. (2019)	Mini-Trampoline Training	0.873 **±** 0.122	0.775 **±** 0.154	0.663 **±** 0.061	0.671 **±** 0.050	12 Weeks
ElDeeb and Abdel-Aziem (2020)	Whole-body vibration	1.030 **±** 0.170	0.920 **±** 0.110	0.710 **±** 0.070	0.640 **±** 0.110	24 Weeks
Rajapakse et al. (2021)	Low-Intensity Vibration	0.959 **±** 0.105	0.961 **±** 0.113	NR	NR	1 Year
Iwamoto et al. (1998)	Physical activity	0.633 **±** 0.090	0.617 **±** 0.060	NR	NR	12 Months
Korpelainen et al. (2006)	Impact exercise	NR	NR	0.670 **±** 0.013	0.663 **±** 0.012	30 Months
Hakestad et al. (2015)	OsteoACTIVE rehabilitation programme	0.943 **±** 0.114	0.968 **±** 0.084	0.795 **±** 0.085	0.777 **±** 0.083	6 Months
Iwamoto et al. (2001)	Exercise training	0.620 **±** 0.087	0.616 **±** 0.044	NR	NR	2 Years
Hettchen et al. (2021)	High-Intensity Exercise	0.873 **±** 0.130	0.904 **±** 0.097	NR	NR	13 Months
Liu-Ambrose et al. (2004)	Agility Training	NR	NR	0.578 **±** 0.146	0.589 **±** 0.133	25 Weeks
Waltman et al. (2022)	Bone-loading exercises	0.885 **±** 0.065	0.885 **±** 0.070	0.706 **±** 0.079	0.683 **±** 0.073	12 Months
Kistler-Fischbacher et al. (2021)	Bone-Targeted Exercise	0.874 **±** 0.018	0.902 **±** 0.017	0.717 **±** 0.012	0.702 **±** 0.012	8 Months
Wang et al. (2015)	Simplified Tai Chi Resistance Training	1.100 **±** 0.170	1.010 **±** 0.130	0.860 **±** 0.120	0.810 **±** 0.100	12 Months

LS, lumbar spine and; FN, femur neck. NR, Not Reported. IG, Intervention group. CG, control group.

**TABLE 4:** Exercise interventions evaluated according to the American College of Sports Medicine’s (ACSM) recommendations.

Author, year	Cardiorespiratory exercise						Resistance exercise								Flexibility exercise						ACSM adherence
	Frequency		Intensity/workload		Duration		Frequency		Intensity/workload		Repetitions		Sets		Frequency		Intensity/workload		Duration		
	4–5/d wk		Moderate intensity, 40%–59% VO^2^R/HRR, CR-10 scale rating of 3–4		20–30 min (up to 45–60 min)		1–2/d wk, gradually increasing to 2–3/d wk		Adjust resistance, medium to high intensity		8–12		1–2		5–7/d wk		Stretch until you feel your muscles being pulled tight or a slight discomfort		Stretching for 10–30 s, repeated 2–4 times		Points (Percent)
Shen et al. (2018)	7	☹	Yi Jin Jing 2 groups	☺	45–60	☺									7	☺	Slight discomfort	☺	NR	☹	9/12 (75%)
Basat et al. (2013)							3	☺	ACSM intensity	☺	10	☺	2	☺							8/8 (100%)
Lin et al. (2022)	2	☹	NR	☹	50	☺									2	☹	NR	☹	NR	☹	5/12 (42%)
Liu et al. (2015)	7	☹	Ba Duan Jin 3 groups	☺	60	☺									7	☺	Straighten and tense	☺	NR	☹	9/12 (75%)
Hartard et al. (1996)							2	☺	70% 1RM	☺	8–12	☺	≥ 2	☺							8/8 (100%)
Hourigan et al. (2008)	2	☹	NR	☹	45	☺	2	☺	NR	☹	NR	☹	NR	☹	NR	☹	NR	☹	5 min	☺	12/20 (60%)
Brentano et al. (2008)							3	☺	45%–80% 1RM	☺	6–20	☹	2–4	☺							7/8 (88%)
Bergström et al. (2008)	4/5	☺	30 min quick walk	☺	55	☺									5	☺	NR	☹	5 min	☺	11/12 (92%)
Bocalini et al. (2010)							3	☺	60%–70% 1RM	☺	10–12	☺	3	☹							6/8 (75%)
Mosti et al. (2013)							3	☺	85%–90% 1RM	☺	3–5	☹	4	☹							4/8 (50%)
Wayne et al. (2012)	3–7	☺	NR	☹	≥30	☺									3–7	☺	NR	☹	NR	☹	9/12 (75%)
Bravo et al. (1996)	3	☹	60%–70% HRR	☹	60	☺	3	☺	Maximum Repeat	☺	12–15	☹	NR	☹							7/14 (50%)
Watson et al. (2018)							2	☺	≥85% 1RM	☺	5	☹	2–5	☺							6/8 (75%)
Watson et al. (2015)							2	☺	80%–85% 1RM	☺	5	☹	2–5	☺							6/8 (75%)
Chien et al. (2000)	3	☹	40%–85% VO^2^R	☹	50	☺															3/6 (50%)
Kienberger et al. (2022)							2	☺	50%–70% 1RM	☺	10–15	☺	3	☹							6/8 (75%)
Cheng et al. (2022)	3	☹	Step speed ≥1.3 m/s	☹	30–60	☺															3/6 (50%)
Nelson et al. (1994)							2	☺	50%–80% 1RM	☺	3–8	☹	3	☹							4/8 (50%)
Bolton et al. (2012)	7	☹	NR	☹	60	☺	3	☺	Medium strength	☹	8–12	☺	2	☺	3	☹	NR	☹	NR	☹	11/20 (55%)
Marchese et al. (2012)	3	☹	NR	☹	60	☺	3	☺	NR	☹	NR	☹	NR	☹							8/14 (57%)
Posch et al. (2019)	2	☹	Balance exercise	☹	45–60	☺	2	☺	NR	☹	NR	☹	NR	☹							7/14 (50%)
ElDeeb and Abdel-Aziem (2020)							2	☺	25–35 Hz	☹	9	☺	NR	☹							6/8 (75%)
Rajapakse et al. (2021)							7	☹	30 Hz	☹	Ind.tail	☹	NR	☹							3/8 (38%)
Iwamoto et al. (1998)	7	☹	Ind.tail	☹	NR	☹	7	☹	NR	☹	15	☹	NR	☹							4/14 (29%)
Korpelainen et al. (2006)	7	☹	NR	☹	45	☺	7	☹	NR	☹	NR	☹	NR	☹							6/14 (43%)
Hakestad et al. (2015)	3	☹	NR	☹	60	☺	3	☺	NR	☹	5–12	☹	2–3	☺							9/14 (64%)
Iwamoto et al. (2001)	7	☹	Ind.tail	☹	NR	☹	7	☹	NR	☹	15	☹	NR	☹							4/14 (29%)
Hettchen et al. (2021)	3	☹	65%-80%HRmax	☹	20	☺	4	☹	Ind.tail	☹	8–16	☺	Ind.tail	☺							5/14 (36%)
Liu-Ambrose et al. (2004)							NR	☹	50%–85% 1RM	☺	10–15	☺	2	☺							7/8 (88%)
Waltman et al. (2022)	3	☹	Ind.tail	☹	NR	☹	3	☺	Ind.tail	☹	8–12	☺	NR	☹							8/14 (57%)
Kistler-Fischbacher et al. (2021)	2	☹	NR	☹	40	☺	2	☺	80%–85% 1RM	☺	5	☹	5	☹							7/14 (50%)
Wang et al. (2015)	4	☺	NR	☹	60	☺									4	☹	Full stretch	☺	10 min	☺	9/12 (75%)

ACSM, American College of Sports Medicine. Ind. tail, individually tailored. NR, not reported. Happy/green face, fulfils recommendation (2 points), neutral/yellow face, uncertain fulfilment (1 point), unhappy/red face, does not fulfil recommendation (0 points).

### 3.3 Risk of bias

**FIGURE 2:**
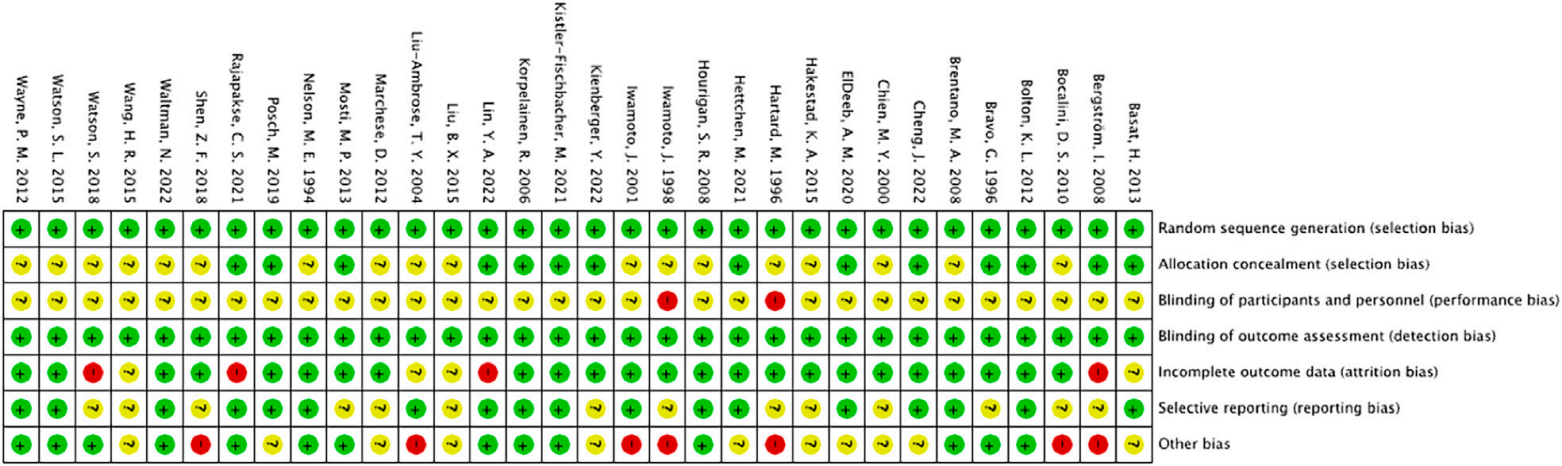
Risk of bias summary: The review author’s judgement of the risk of bias of each included study.

All studies were considered to have low risk of bias for random sequence generation. Among the 32 studies, 14 were considered to have low risk of bias for allocation concealment, while 18 did not report their allocation method and were therefore considered to have unclear risk. The risk of bias for blinding assessment was relatively high, as exercise interventions were difficult to implement under double-blind conditions, resulting in an overall higher risk of bias for this indicator. For outcome blinding assessment, as the outcome measure in this study was BMD measured using dual-energy X-ray absorptiometry or dual-photon absorptiometry (DPA), the risk of bias was considered low. In incomplete outcome reporting, 9 studies had a discrepancy in the number of post-intervention participants compared to the baseline, 5 studies had a small number of dropouts (<10 participants) and were therefore considered to have some risk, and 4 studies had a large difference (≥10 participants) between the number of participants before and after the intervention, and were therefore considered to have a high risk. The risk of selective reporting bias was considered low for 18 studies, and moderate for 14 studies, as they did not report their pre-registered plan or did not provide a detailed explanation for participant dropouts. Seven studies had other risk of bias (Figure 2).

### 3.4 The impact of adherence to ACSM recommendations on lumbar spine and femoral neck BMD

Adherence with the ACSM recommendations was ≥ 70% in 14 studies and < 70% in 18 studies (Table 4). The reasons for low adherence were partly due to exercise interventions not matching the ACSM recommendations in terms of dose, and partly due to insufficient information provided in the studies to allow for appropriate evaluation of exercise prescription.

#### 3.4.1 Lumbar spine BMD

When the study results were based on lumbar spine BMD, a total of 27 studies involving 1,539 participants were included. Among them, 11 studies demonstrated high adherence to ACSM recommendations, while 16 studies had low or uncertain adherence. Initially, a heterogeneity test was conducted, revealing an I^2^ value greater than 50% (I^2^ = 79.9%). Therefore, a random-effects model was used for statistical analyses.

Data analyses indicated that the overall effect of exercise on lumbar spine BMD was 0.15 (95% CI: −0.09, 0.39). This suggests that the effect size of exercise on lumbar spine BMD is relatively small in the overall sample, and the confidence interval spans zero. This indicates that it is inconclusive whether exercise has a significant impact on lumbar spine BMD.

Further subgroup analyses revealed the following results: In the subgroup with high adherence to ACSM recommendations, the SMD was 0.31 (95% CI: 0.01, 0.62), with a heterogeneity of 64.8%. This indicates that exercise with high adherence to ACSM recommendations may have a positive impact on lumbar spine BMD, and the confidence interval of the effect does not include zero, demonstrating statistical significance. However, moderate heterogeneity suggests some degree of variability among these studies. In the subgroup with low or uncertain adherence to ACSM recommendations, the SMD was 0.04 (95% CI: −0.29, 0.37), with a heterogeneity of 83.6%. This suggests that exercise with low or uncertain adherence to ACSM recommendations may not have a significant impact on lumbar spine BMD, as the confidence interval of the effect spans zero.

**FIGURE 3:**
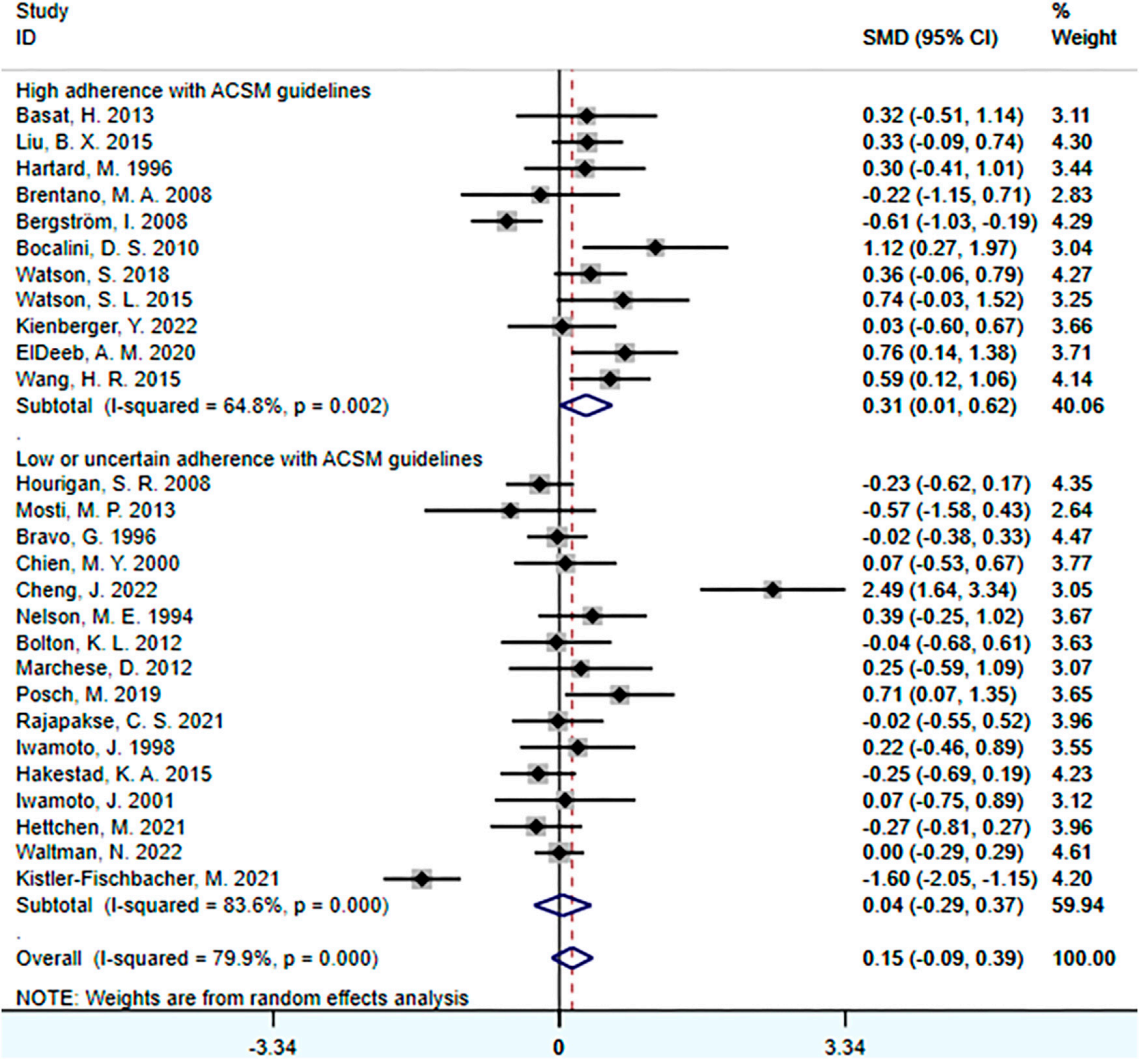
Forest plot for meta-analyses of the effect of exercise on bone mineral density of the lumbar spine in individuals with OP.

Overall, compared to exercise with low or uncertain adherence to ACSM recommendations (SMD: 0.31 > 0.04), exercise with high adherence to ACSM recommendations tends to have a more positive relationship with lumbar spine BMD. However, due to the overall high heterogeneity and differences among subgroups with different adherence levels, this may be attributed to factors such as study design, sample characteristics, and intervention measures **(** Figure 3 **)**.

**FIGURE 4:**
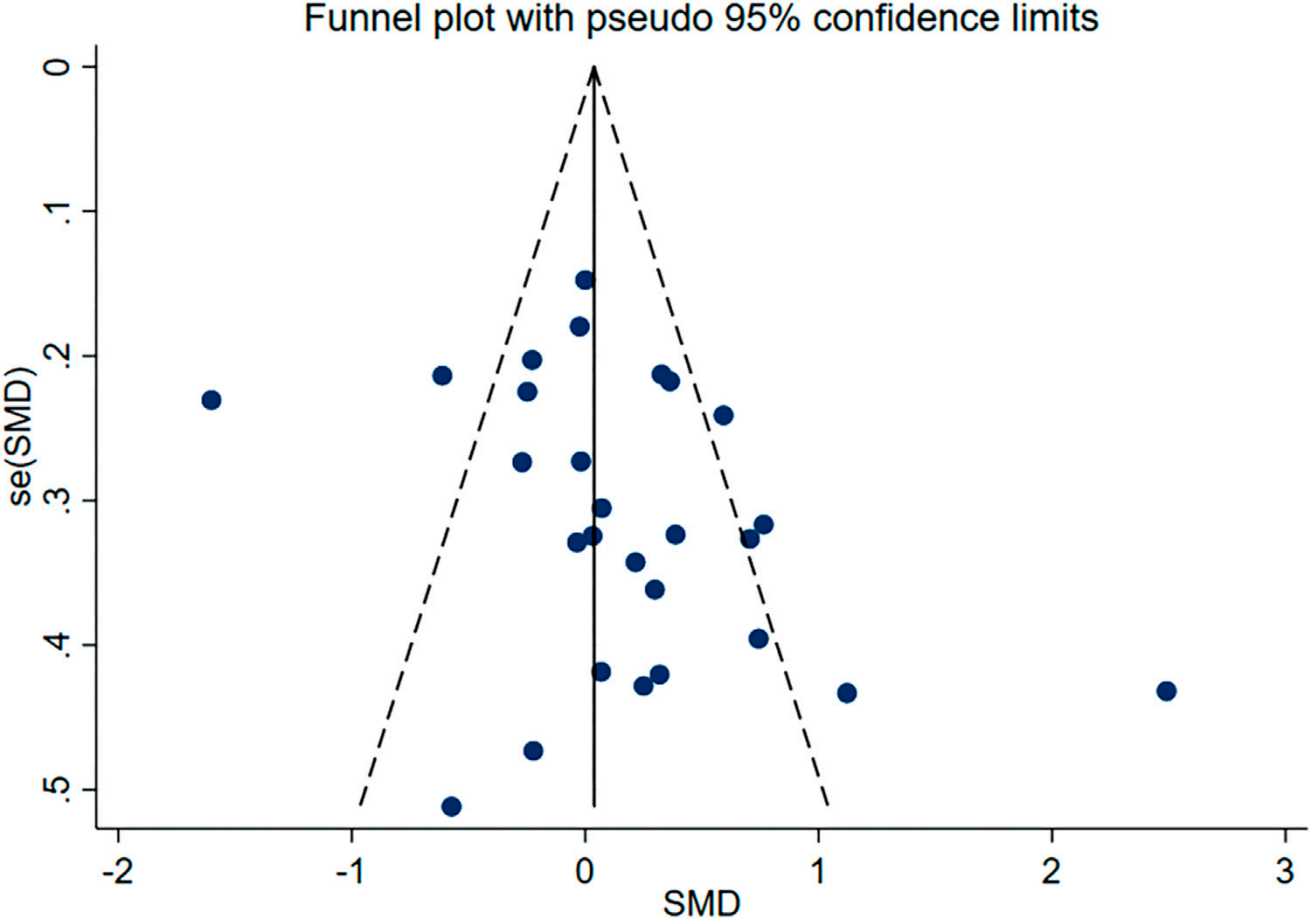
Funnel plot containing lumbar spine bone density study.

**FIGURE 5:**
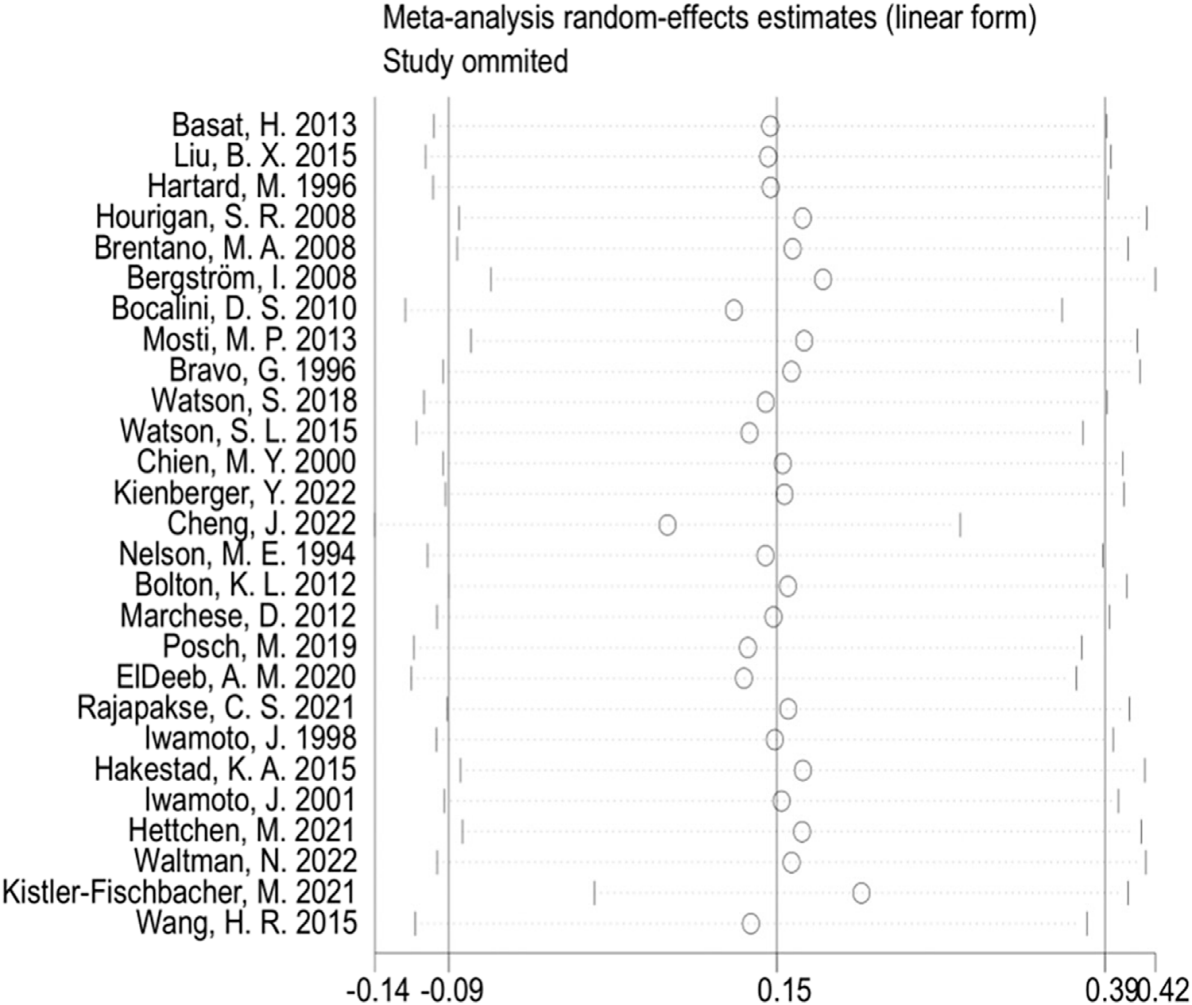
Sensitivity analyses (lumbar spine BMD).

Subsequently, we conducted publication bias tests and sensitivity analyses. The visual inspection of the funnel plot **(** Figure 4 **)** revealed approximate symmetry on both sides, indicating the absence of obvious publication bias. Furthermore, we conducted Begg’s test (*p* = 0.118) and Egger’s test (*p* = 0.114), which further supported the absence of significant publication bias. In the sensitivity analyses, performed by sequentially excluding individual studies **(** Figure 5 **)**, we found that no single study had a substantial impact on the overall results, suggesting the robustness of the findings.

#### 3.4.2 Femoral neck BMD

When the study reports were based on femoral neck BMD, a total of 23 studies involving 1,606 participants were included. Among them, 12 studies demonstrated high adherence to ACSM recommendations, while 11 studies had low or uncertain adherence. Initially, a heterogeneity test was conducted, revealing an I^2^ value greater than 50% (I^2^ = 68.7%). Therefore, a random-effects model was used for statistical analyses.

Regarding the overall effect, the overall effect of exercise on femoral neck BMD was 0.36 (95% CI: 0.18, 0.55). This indicates that exercise has a relatively large effect on femoral neck BMD in the overall sample, and the confidence interval does not include zero. This suggests that exercise has a significant positive impact on femoral neck BMD.

Further analyses of the subgroup results revealed that in the subgroup with high adherence to ACSM recommendations, the SMD was 0.45 (95% CI: 0.20, 0.70), with a heterogeneity of 55.8%. This indicates that exercise with high adherence to ACSM recommendations may have a larger positive impact on femoral neck BMD, and the confidence interval of the effect does not include zero, indicating statistical significance. The relatively low heterogeneity suggests a higher consistency among these studies and more reliable results. In the subgroup with low or uncertain adherence to ACSM recommendations, the SMD was 0.28 (95% CI: 0.00, 0.56), with a heterogeneity of 77%. This suggests that exercise with low or uncertain adherence to ACSM recommendations may still have a certain degree of positive impact on femoral neck BMD, as the confidence interval of the effect does not include zero. However, the higher heterogeneity indicates significant differences among these studies, and therefore, the results need to be interpreted with caution.

**FIGURE 6:**
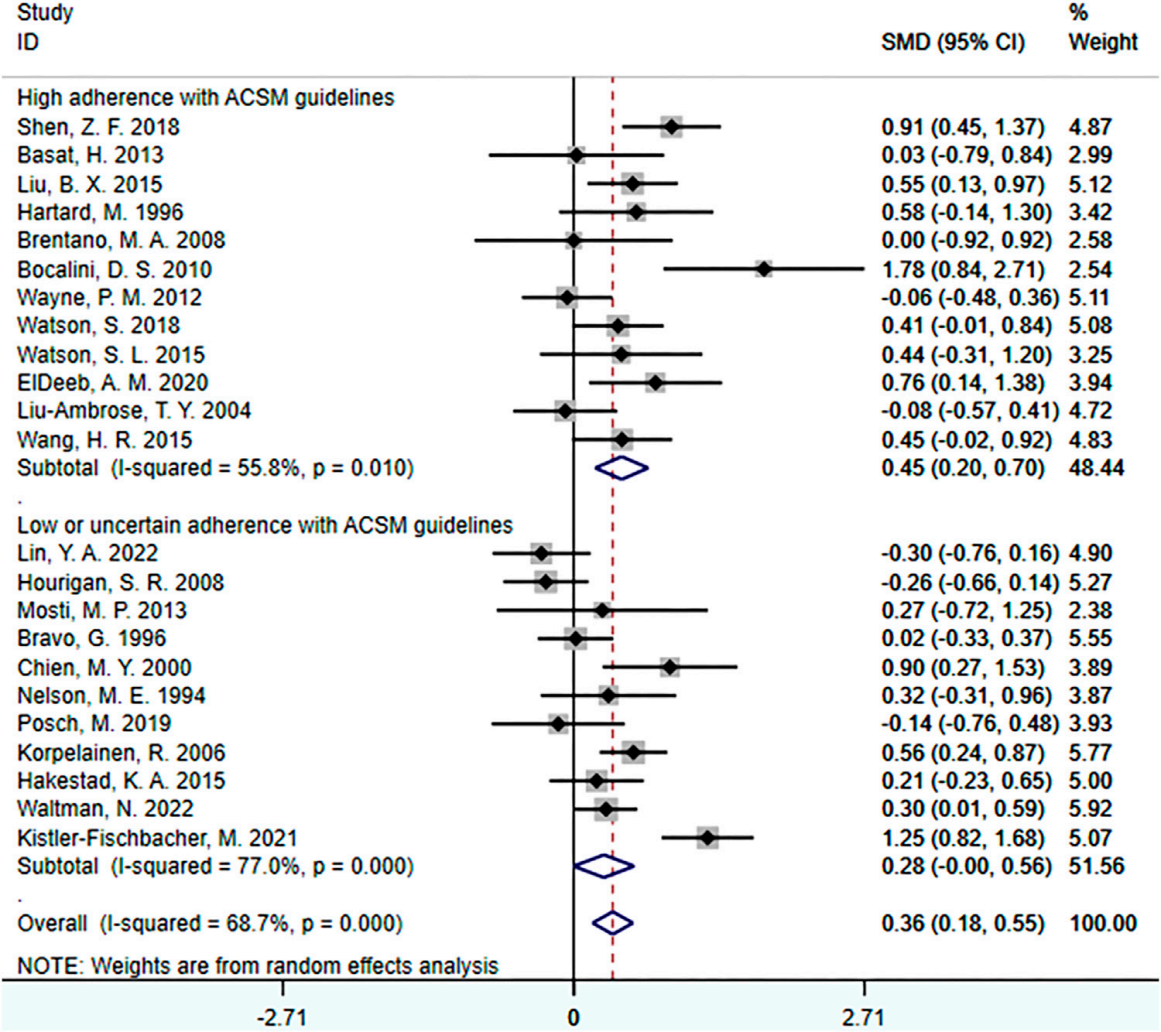
Forest plot for meta-analyses of the effect of exercise on femoral neck bone mineral density in individuals with OP.

In summary, exercise has a relatively large and statistically significant effect on femoral neck BMD. Further subgroup analyses reveals that compared to exercise with low or uncertain adherence to ACSM recommendations (SMD: 0.45 > 0.28), interventions with high adherence to ACSM recommendations may have a larger positive impact on femoral neck BMD, and the results are statistically significant (Figure 6).

**FIGURE 7:**
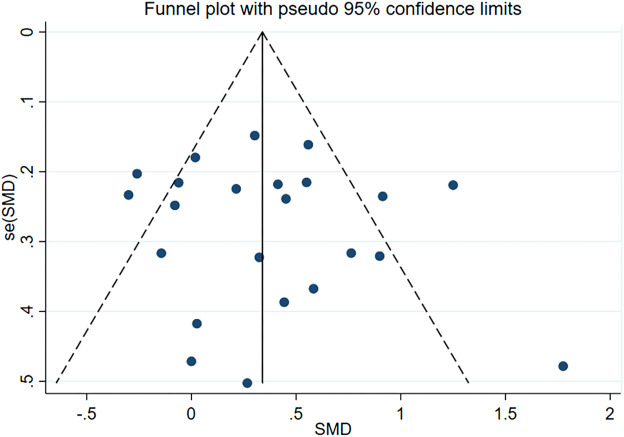
Funnel plot containing femoral neck bone density study.

**FIGURE 8:**
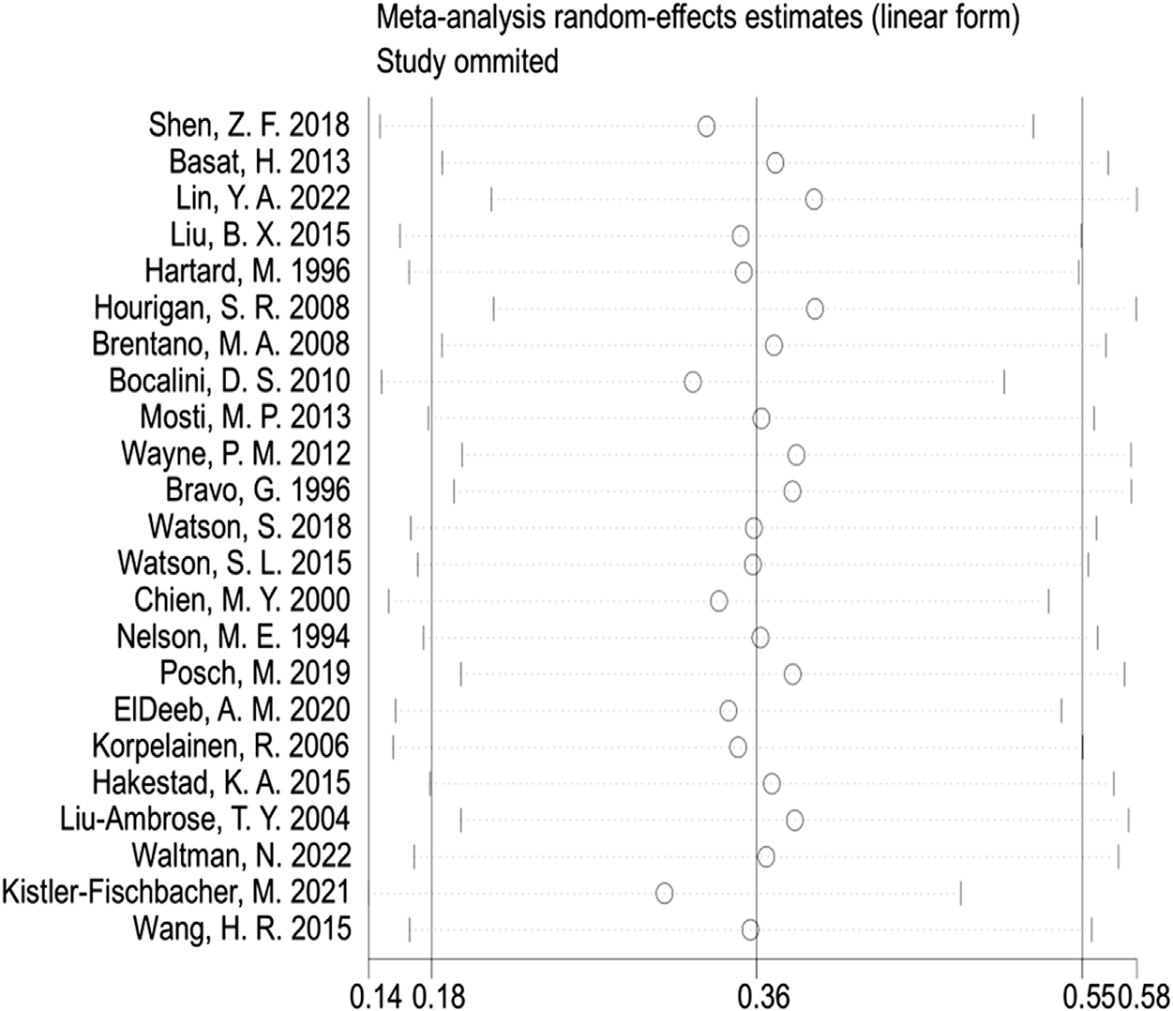
Sensitivity analyses (femur neck BMD).

In the publication bias tests and sensitivity analyses, we found that the funnel plot exhibited approximate symmetry on both sides (Figure 7), indicating the absence of obvious publication bias. Furthermore, we conducted Begg’s test (*p* = 0.509) and Egger’s test (*p* = 0.602), which once again supported the absence of significant publication bias. In the sensitivity analyses, performed by sequentially excluding individual studies (Figure 8), we found that no single study had a substantial impact on the overall results, indicating the robustness of the findings.

### 3.5 The impact of ACSM adherence on lumbar spine and femoral neck BMD in resistance exercise

We further investigated the impact of ACSM adherence on lumbar spine and femoral neck BMD in resistance exercise.

#### 3.5.1 Lumbar spine BMD

When the study results were based on lumbar spine BMD, a total of 20 studies were included, with 1,140 participants. Among them, 11 studies demonstrated high adherence to ACSM recommendations, while 9 studies had low or uncertain adherence. Initially, a heterogeneity test was conducted, revealing an I^2^ value of 74.4%. Therefore, a random-effects model was used for statistical analyses.

Regarding the overall effect, the SMD was 0.02 (95% CI: −0.22, 0.27). This indicates that in the overall sample, the effect size of resistance exercise on lumbar spine BMD is relatively small, and the confidence interval includes zero. This suggests that it is uncertain whether resistance exercise has a significant impact on lumbar spine BMD.

**FIGURE 9:**
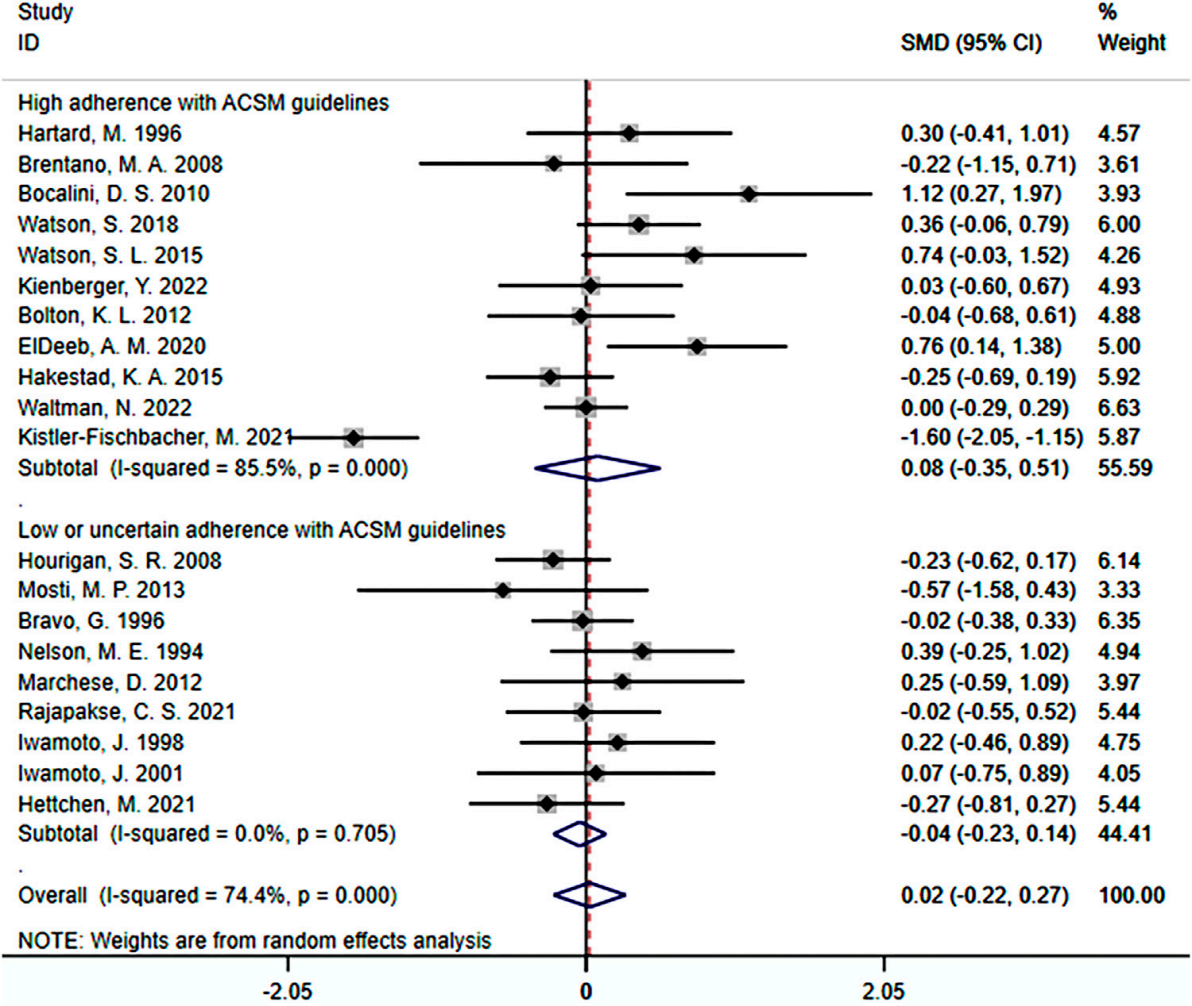
Forest plot of the effect of ACSM adherence on lumbar spine BMD in resistance exercise.

Further analyses of subgroup results: In the subgroup with high adherence to ACSM recommendations, the SMD was 0.08 (95% CI: −0.35, 0.51), with a heterogeneity of 85.5%. This indicates that resistance exercise with high adherence to ACSM recommendations may have some positive impact on the target outcome. However, the confidence interval of the effect includes zero, indicating uncertainty in the statistical significance of the effect. The high heterogeneity suggests significant differences among these studies. In the subgroup with low or uncertain adherence to ACSM recommendations, the SMD was −0.04 (95% CI: −0.23, 0.14), with a heterogeneity of 0%. This indicates that resistance exercise with low or uncertain adherence to ACSM recommendations may not have a significant impact on lumbar spine BMD. The confidence interval includes zero, indicating that the statistical significance does not support a positive impact of resistance exercise with low ACSM adherence on lumbar spine BMD. Moreover, the 0% heterogeneity suggests high consistency among these studies (Figure 9).

In summary, the effect of resistance exercise on lumbar spine BMD is relatively small and not statistically significant. However, there may be some positive effects on lumbar spine BMD with resistance exercise that adheres to ACSM recommendations, although the statistical significance remains uncertain. On the other hand, resistance exercise with low or uncertain adherence to ACSM recommendations may not have a significant impact. These results highlight the potential differences in the effects of ACSM recommendations across different subgroups, and the high heterogeneity suggests substantial variation among the studies. However, it is important to note that the confidence interval of the overall effect includes zero, indicating uncertainty regarding the effect of ACSM on the target outcome in the overall sample. This uncertainty may be influenced by factors such as study design, sample characteristics, or other factors. Therefore, these results should be interpreted with caution, and further research is recommended to gain a comprehensive understanding of the impact of ACSM and to determine its role in different populations.

In the publication bias test and sensitivity analyses, we found that the funnel plot is roughly symmetrical on both sides, indicating no obvious publication bias (Supplementary Appendix SA2). Furthermore, the Begg test (*p* = 0.436) and Egger test (*p* = 0.258) provided additional evidence of no significant publication bias. Sensitivity analyses, by systematically excluding individual studies, did not reveal any single article significantly influencing the overall results, suggesting the robustness of the findings (Supplementary Appendix SA3).

#### 3.5.2 Femoral neck BMD

When the study results focus on femoral BMD, there are a total of 20 studies with 1,140 participants, including 11 studies with high adherence to ACSM recommendations and 6 studies with low or uncertain adherence. Firstly, heterogeneity test indicated an I^2^ value of 67.4%, suggesting the use of a random-effects model for statistical analyses.

The meta-analyses revealed that the overall effect of resistance exercise on femoral neck BMD was 0.36 (95% CI: 0.14, 0.58). This indicates a relatively large effect size of resistance exercise on femoral neck BMD, and the confidence interval does not include zero. These findings suggest a significant positive impact of resistance exercise on femoral neck BMD.

**FIGURE 10:**
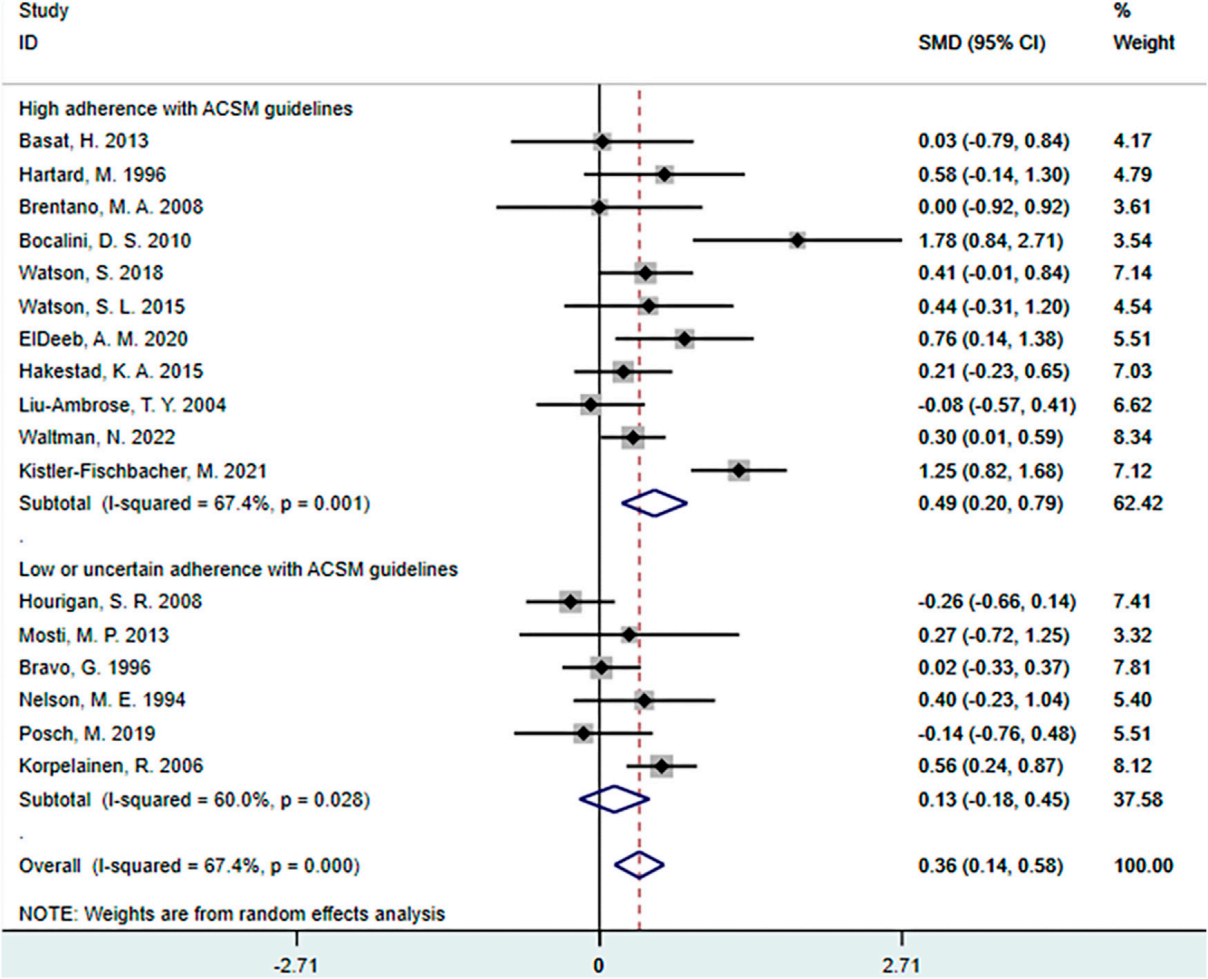
Forest plot of the effect of ACSM adherence on femoral neck BMD in resistance exercise.

Further subgroup analyses showed that in the high adherence subgroup to ACSM recommendations, the SMD was 0.49 (95% CI: 0.20, 0.79), with a heterogeneity of 67.4%. This indicates a substantial positive impact of high adherence to ACSM-guided exercise on femoral neck BMD, and the confidence interval does not include zero, indicating statistical significance. The level of heterogeneity is similar to the overall effect, indicating low consistency among these studies. In the low or uncertain adherence subgroup to ACSM recommendations, the SMD was 0.13 (95% CI: −0.18, 0.45), with a heterogeneity of 60%. This suggests that ACSM may have a certain degree of positive impact on the target outcome in the low or uncertain adherence group, as the confidence interval spans zero. The level of heterogeneity indicates higher consistency among these studies (Figure 10).

Overall, resistance exercise has a relatively large and statistically significant effect on femoral neck BMD. High adherence to ACSM-guided resistance exercise has a substantial positive impact on femoral BMD, and the results are statistically significant. Even in low or uncertain adherence to ACSM-guided resistance exercise, there may still be a certain degree of positive impact on the target outcome. However, comparing the SMD differences between high adherence to ACSM and low or uncertain adherence (SMD: 0.49 > 0.13), high adherence to ACSM-guided resistance exercise has a more positive effect on femoral neck BMD than low or uncertain adherence.

In the publication bias test and sensitivity analyses, we found that the funnel plot is roughly symmetrical on both sides, indicating no obvious publication bias (Supplementary Appendix SA4). Furthermore, the Begg’s test (*p* = 0.621) and Egger’s test (*p* = 0.517) provided additional evidence that there is no significant publication bias. Sensitivity analyses, conducted by removing individual studies one by one, showed that no single study significantly influenced the overall results, indicating the robustness of the findings (Supplementary Appendix SA5).

### 3.6 Meta-regression analyses

Based on the observed high heterogeneity, we conducted a multiple-factor meta-regression analyses to explore potential research characteristics that may influence heterogeneity. We considered participant characteristics (such as individual characteristics related to health status, gender, age, etc.), intervention features (ACSM adherence, intervention duration, etc.), and outcome features. Regarding participant characteristics, we selected studies that excluded participants with other metabolic diseases and excluded them as factors in the meta-regression analyses. Regarding gender, we observed that only 3 out of 32 studies included male participants, accounting for 3.3% of the total sample size. Due to the significant gender imbalance, we decided not to include gender as one of the factors in the meta-regression analyses. In terms of participant age, the majority of participants were postmenopausal women with minimal age differences, and some studies did not report participant ages. This posed challenges for conducting the meta-regression analyses, and thus we excluded age as a factor. In the outcome features, BMD was measured using dual-energy X-ray, and therefore it was not included in the discussion factors for the meta-regression analyses. Finally, we focused on sample size, publication year, country, intervention duration, and ACSM adherence as potential factors influencing heterogeneity in the meta-regression analyses.

The results of the multiple-factor meta-regression analyses for lumbar spine BMD indicate that, after adjusting for covariates such as sample size, publication year, country, intervention duration, ACSM adherence, and total _cons, we did not find any significant associations between these variables and lumbar spine BMD (sample size: *p* = 0.180, publication year: *p* = 0.737, country: *p* = 0.609, intervention duration: *p* = 0.661, ACSM adherence: *p* = 0.500, _cons: *p* = 0.761). Therefore, based on our analyses, these factors do not have a statistically significant impact on lumbar spine BMD **(** Table 5 **)**.

**TABLE 5:** Univariate meta-regression analysis of the impact of different study characteristics on inter-study heterogeneity.

Outcome	Covariates	Regression coefficient	Standard error	t	P > |t|	[95% Conf. Interval]
Lumbar Spine	Sample size	−0.0050319	0.0036276	−1.39	0.180	[−0.0125759, 0.0025121]
	Year published	0.0054404	0.0159849	0.34	0.737	[−0.0278019, 0.0386828]
	Country	−0.0195674	0.0376951	−0.52	0.609	[−0.0979587, 0.0588239]
	Length of intervention	−0.0034295	0.0077008	−0.45	0.661	[−0.0194442, 0.0125851]
	ACSM Adherence	−0.1936663	0.2824323	−0.69	0.500	[−0.7810164, 0.3936839]
	_cons	−9.936236	32.23634	−0.31	0.761	[−76.97538, 57.10291]
Femoral Neck	Sample size	−0.0037749	0.0036159	−1.04	0.311	[−0.0114038, 0.0038541]
	Year published	0.0098027	0.0140524	0.70	0.495	[−0.0198453, 0.0394507]
	Country	−0.0273973	0.0237037	−1.16	0.264	[−0.0774077, 0.0226132]
	Length of intervention	0.0094046	0.0058451	1.61	0.126	[−0.0029275, 0.0217367]
	ACSM Adherence	−0.0679127	0.2329978	−0.29	0.774	[−0.5594951, 0.4236696]
	_cons	−19.16286	28.34522	−0.68	0.508	[−78.96606, 40.64033]

Similarly, the results of the multiple-factor meta-regression analyses for femoral neck BMD show that, after adjusting for covariates such as sample size, publication year, country, intervention duration, ACSM adherence, and total _cons, we did not find any significant associations between these variables and femoral neck BMD (sample size: *p* = 0.311, publication year: *p* = 0.495, country: *p* = 0.264, intervention duration: *p* = 0.126, ACSM adherence: *p* = 0.774, _cons: *p* = 0.508). Therefore, according to our analyses, these factors do not have a statistically significant impact on femoral neck BMD **(** Table 5 **)**.

In conclusion, based on the results of the multiple-factor meta-regression analyses, as well as considering the subgroup analyses of ACSM adherence, we did not find statistically significant associations between sample size, publication year, country, intervention duration, ACSM adherence, and BMD of the lumbar spine and femoral neck (*p* > 0.05). This suggests that these factors may not be important in explaining the variability in lumbar spine BMD and femoral neck BMD.

## 4 Discussion

This systematic review and meta-analyses examined the effects of high adherence to the ACSM recommendations versus low or uncertain adherence on lumbar spine and femoral neck BMD in individuals with OP through 32 studies involving 2,005 participants with OP or low bone mass.

### 4.1 Positive effects of exercise intervention on BMD

Through meta-analyses, we found that exercise intervention can improve the BMD of the lumbar spine and femur in individuals with OP. This finding is consistent with common knowledge and previous research conclusions (Consensus development conference: Diagnosis, prophylaxis, and treatment of osteoporosis, 1993; Ernst, 1998), confirming the effectiveness of exercise as a non-pharmacological treatment for individuals with OP. However, previous research summaries have shown that not all exercise interventions can increase the bone density of individuals with OP. And the effect of exercise on BMD varies across different bone locations.

There is evidence to suggest that resistance exercise is superior to aerobic exercise in increasing bone density, but the effects of pure resistance exercise or aerobic exercise on bone density improvement are not significant (Benedetti et al., 2018). Therefore, it may be necessary to develop exercise programs that incorporate multiple types of exercises. From a few analyses on the impact of resistance exercise on BMD, it is evident that there are significant differences in the effects (Kelley et al., 2001; Bemben and Bemben, 2011). Additionally, studies have indicated that exercise intensity and frequency are factors that need to be considered. If exercise intensity or frequency is not properly managed, the results may be meaningless (Chahal et al., 2014; Kemmler and von Stengel, 2014), and excessive physical training can even have unexpected negative effects on bones. A study in 2015, based on extensive experimental and review research, pointed out that different exercise modalities should be adopted in different age groups or populations. Weight-bearing exercise during childhood can prevent OP, while in older adults, emphasis should be placed on strength and balance training. For individuals with arthritis, strength training alone or intensified physical exercise may not yield significant effects; the ideal approach is to combine aerobic exercise with weight-bearing training (Pedersen and Saltin, 2015).

Therefore, the factors influencing the effect of exercise intervention on bone density are not limited to resistance exercise or aerobic exercise alone. Considerations such as exercise modality, intensity, type, and dose are also crucial. Research has shown that exercise modalities such as vibration training and strength training have advantages over activities such as walking or jogging (Nelson et al., 1994; Kelley et al., 2001; Cheng et al., 2022; Kienberger et al., 2022), and whole-body exercises like Tai Chi and Ba Duan Jin have positive effects (Liu and Wang, 2017; Mu et al., 2018; Sun et al., 2022). High-intensity resistance and impact training are more effective than moderate or low-intensity exercise (Kitagawa et al., 2022). However, there is a lack of research on exercise dose, and we are uncertain about the appropriate exercise dose for treating individuals with OP. Exercise dose assessment involves multiple indicators such as exercise intensity, frequency, and duration. Our study found that, according to the recommendations of the ACSM, a high level of adherence to exercise dose is more advantageous for improving BMD compared to low or uncertain adherence.

### 4.2 Exercise adherence according to ACSM recommendations

In ACSM high adherence exercise interventions, various exercise modalities or types are involved, including Yi Jin Jing, strength training, Ba Duan Jin, Tai Chi, resistance training, aerobic exercise, whole-body vibration, agility exercises, and more. Similarly, in ACSM low or uncertain adherence, exercise modalities or types include Taekwondo, balance and weight-bearing exercises, strength training, walking, aerobic exercise, resistance training, and others. This analyses avoids the influence of dominant exercise modalities on the differences in ACSM adherence.

This study’s strength lies in the integration of various exercise modalities, exercise intensities, exercise durations, and other indicators utilized in previous research. It uses ACSM adherence as a grouping factor to validate the effect of exercise dose on improving BMD in individuals with OP. The key point of this study lies in the interpretation of ACSM adherence.

However, currently, the descriptions of exercise dose in RCTs for individuals with OP are not comprehensive enough or can only be attributed to one type of exercise intervention. This makes it difficult for us to assess exercise protocols that incorporate all three types of exercises. Based on this, we conducted a study specifically focusing on the impact of ACSM adherence on BMD within individual exercise types. Initially, our design included studies on aerobic exercise, resistance exercise, and a combination of aerobic and resistance exercises. However, in the final analyses, we encountered significant discrepancies in the number of studies categorized under aerobic exercise and a combination of aerobic and resistance exercises, as well as a limited number of studies overall. These issues made it challenging to support our analyses. Therefore, we conducted an evaluation and analyses specifically for the resistance exercise type regarding ACSM adherence.

Additionally, some studies either did not report or inadequately reported the dose of exercise interventions, describing it only as “individually tailored.” This means that even if the exercise dose of an intervention aligns with high adherence according to ACSM recommendations, it could be incorrectly classified as low or uncertain adherence. For this, we utilized a scoring assessment (0–2) to minimize biases resulting from the lack of certain information elements as much as possible.

### 4.3 Effect of ACSM adherence on the effectiveness of exercise intervention

T. Moseng et al. assessed the impact of adherence to ACSM recommendations on pain and physical function in patients with knee osteoarthritis, based on ACSM-recommended exercise dose for arthritis patients. The systematic review demonstrated that exercise interventions highly adherent to ACSM recommendations showed significant improvements in hip osteoarthritis patients compared to interventions with uncertain adherence (Moseng et al., 2017). Another study also evaluated the level of adherence to exercise guidelines based on ACSM recommendations and examined the effects of ACSM interventions versus non-ACSM interventions on muscle strength in patients with knee osteoarthritis. The results favored ACSM interventions, indicating that ACSM interventions were more beneficial for increasing muscle strength in knee osteoarthritis patients (Bartholdy et al., 2017). One key difference between the two studies was that the assessment of ACSM adherence in T. Moseng et al.’s study involved an overall evaluation of three types of exercises without further differentiation, while C. Bartholdy et al.’s study specifically evaluated the adherence to ACSM recommendations in resistance exercise or strength training. However, both studies yielded consistent results, indicating that exercise interventions with higher adherence to ACSM recommendations have better outcomes.

In our study, we attempted to include as many types of exercises as possible to assess the impact of ACSM adherence on intervention effectiveness. In the meta-analyses, by conducting subgroup analyses based on the adherence of exercise dosage to ACSM recommendations, we found that exercise interventions with high ACSM adherence were more effective in improving lumbar spine and femoral neck BMD in individuals with OP compared to exercises with low or uncertain ACSM adherence (SMD: 0.31 vs. 0.04 and 0.45 vs. 0.28). Furthermore, the improvement in femoral neck BMD was greater than that in lumbar spine BMD (SMD: 0.45 vs. 0.31).

When analyzing the impact of ACSM adherence on lumbar spine BMD within resistance exercise, although the overall SMD and subgroup SMD did not show a significant effect of exercise on lumbar spine BMD, there was still a slight advantage for exercise with high ACSM adherence compared to exercise with low or uncertain ACSM adherence. However, the interpretation of these results should be cautious, and further research is needed to validate these findings. In contrast, the impact of ACSM adherence on femoral neck BMD yielded clearer results. Resistance exercise with high ACSM adherence had a significantly higher SMD compared to exercise with low or uncertain ACSM adherence (SMD: 0.49 vs. 0.13). This suggests that compared to studies with low or uncertain ACSM adherence, resistance exercise with high ACSM adherence has a more positive effect on femoral neck BMD.

In summary, according to multiple studies, exercise that adheres to ACSM recommendations has a positive effect on BMD. However, some exercise interventions that do not comply with ACSM recommendations have shown high positive effects in certain studies, and sometimes even better than adherence to ACSM recommendations. Although we conducted strict evaluations and analyses, this method still conceals the influence of adherence differences, which may have a more favorable or unfavorable impact on our results. Therefore, based on research results, recommended exercise dose by ACSM has a positive effect on BMD, but can only provide a general framework. Similar to drug treatment, different exercise dose should be chosen according to the characteristics of different subjects during practical implementation.

### 4.4 Other factors affecting the effect of exercise on BMD

Firstly, like pharmacological treatment, exercise therapy is also being explored as a way to manage individuals with OP. In the process of determining the optimal exercise dose, it is important to provide detailed descriptions of the exercise prescription for interventions to precisely identify the reasonable range of dose. Moreover, the heterogeneity among studies increases the difficulty of exploring the optimal exercise program and dose. Secondly, based on current knowledge, we can only infer that exercise itself can improve BMD in individuals with OP, and there is no specific exercise program that has been proven to be superior to others. Our current research still focuses on comparing the effects of specific aspects of exercise interventions and lacks clear standardized recommendations to guide the determination of exercise programs and dose. The ACSM provides recommended guidelines, but there are still controversies and uncertainties. Our study demonstrates the feasibility of ACSM exercise programs and dose.

However, there was relatively high heterogeneity among the studies, and we explored the sources of this heterogeneity through a multiple-factor meta-regression. We did not find any statistically significant associations between sample size, publication year, country, intervention duration, and ACSM adherence with bone density (*p* > 0.05). This suggests that these factors may not be important in explaining the variability in lumbar spine BMD and femoral neck BMD.

Highly adherent exercise interventions, according to ACSM recommendations, include various types of exercises. Additionally, the interventions provided varied in terms of frequency, intensity, and duration, making it challenging to compare and recommend general standards for the optimal exercise interventions. Previous meta-analyses attempted to group studies based on other factors to determine if there are better exercise intervention approaches, but the significant differences in the structure of exercise interventions across multiple studies can obscure the relationship with treatment effects. This lack of precision in determining and designing the optimal exercise program for individuals with OP, including the type, dose, and duration, poses challenges.

Determining the optimal exercise program and dose is a complex task that requires considering individual differences, various types of exercises, exercise intensity and frequency, and personalization factors. Future research and practice should focus on establishing more specific recommendations and individualized exercise prescriptions to help individuals achieve the best possible improvement in bone density.

### 4.5 Limitations of the study and future prospects

Despite the valuable findings and contributions of this study, there are certain limitations that should be acknowledged. Firstly, the study relied on a meta-analyses of existing research, which means it is subject to the limitations and biases present in the original studies. Additionally, the heterogeneity among the included studies in terms of sample size, study design, and outcome measures may have influenced the overall results and interpretations. Another limitation is the lack of standardized protocols for exercise interventions and dose in the included studies. The variability in exercise types, intensities, durations, and frequencies makes it challenging to draw definitive conclusions about the optimal exercise dose for improving BMD in individuals with OP. Future research should focus on developing standardized protocols and recommendations for exercise interventions in this population. Thirdly, previous studies and our own research both used a mixed method that combined different types of exercise to calculate adherence to ACSM recommendations. However, this approach has inherent limitations and may weaken the practical significance in exercise practice. Future studies could consider assigning different weights to the effects of different exercise types on bone mineral density or evaluating the influence of adherence to a single ACSM indicator on the effectiveness of exercise outcomes. Finally, the study primarily focused on the impact of exercise on BMD and did not thoroughly explore other relevant outcomes such as fracture risk, quality of life, or functional abilities. Future studies should consider a broader range of outcomes to provide a comprehensive understanding of the effects of exercise interventions on individuals with OP.

In terms of future prospects, it is crucial to conduct well-designed randomized controlled trials with larger sample sizes and longer follow-up periods. These studies should incorporate standardized exercise protocols and consider individual characteristics such as age, gender, and comorbidities to tailor exercise interventions effectively. Moreover, the integration of emerging technologies and interventions, such as virtual reality-based exercises, wearable devices for monitoring physical activity, and novel exercise modalities, may offer new opportunities to enhance the effectiveness of exercise interventions in OP management. In conclusion, while this study sheds light on the relationship between exercise interventions and BMD in individuals with OP, it is important to acknowledge its limitations and address them in future research. By overcoming these limitations and exploring new avenues, we can further advance our understanding and optimize exercise interventions for the management of OP.

## 5 Conclusion

This review supports the recommendation of exercise as an effective means of improving BMD in individuals with OP, and our results once again confirm this conclusion. When exploring the optimal exercise dose for individuals with OP, we found that exercise interventions with high adherence to the ACSM recommendations were more effective in improving BMD in the lumbar spine and femur neck compared to interventions with low or uncertain adherence to ACSM. However, since some studies did not provide detailed exercise intervention protocols, future research will require more experimental designs and larger samples for validation.

## Data Availability

The original contributions presented in the study are included in the article/Supplementary Materials, further inquiries can be directed to the corresponding author.

## References

[B1] Bartholdy C. Juhl C. Christensen R. Lund H. Zhang W. Henriksen M. (2017). The role of muscle strengthening in exercise therapy for knee osteoarthritis: A systematic review and meta-regression analysis of randomized trials. Semin. Arthritis Rheum. 47, 9–21. 10.1016/j.semarthrit.2017.03.007 28438380

[B2] Basat H. Esmaeilzadeh S. Eskiyurt N. (2013). The effects of strengthening and high-impact exercises on bone metabolism and quality of life in postmenopausal women: A randomized controlled trial. J. Back Musculoskelet. Rehabil. 26, 427–435. 10.3233/bmr-130402 23948830

[B3] Bemben D. A. Bemben M. G. (2011). Dose–response effect of 40 weeks of resistance training on bone mineral density in older adults. Osteoporos. Int. 22, 179–186. 10.1007/s00198-010-1182-9 20195844

[B4] Benedetti M. G. Furlini G. Zati A. Letizia Mauro G. (2018). The effectiveness of physical exercise on bone density in osteoporotic patients. Biomed. Res. Int. 2018, 4840531. 10.1155/2018/4840531 30671455 PMC6323511

[B5] Bergström I. Landgren B. Brinck J. Freyschuss B. (2008). Physical training preserves bone mineral density in postmenopausal women with forearm fractures and low bone mineral density. Osteoporos. Int. 19, 177–183. 10.1007/s00198-007-0445-6 17768587

[B6] Bocalini D. S. Serra A. J. Dos Santos L. (2010). Moderate resistive training maintains bone mineral density and improves functional fitness in postmenopausal women. J. Aging Res. 2010, 760818. 10.4061/2010/760818 21188230 PMC3003976

[B7] Bolton K. L. Egerton T. Wark J. Wee E. Matthews B. Kelly A. (2012). Effects of exercise on bone density and falls risk factors in post-menopausal women with osteopenia: A randomised controlled trial. J. Sci. Med. Sport 15, 102–109. 10.1016/j.jsams.2011.08.007 21996058

[B8] Bonura F. (2009). Prevention, screening, and management of osteoporosis: An overview of the current strategies. Postgrad. Med. 121, 5–17. 10.3810/pgm.2009.07.2021 19641263

[B9] Bravo G. Gauthier P. Roy P. Payette H. Gaulin P. Harvey M. (1996). Impact of a 12-month exercise program on the physical and psychological health of osteopenic women. J. Am. Geriatr. Soc. 44, 756–762. 10.1111/j.1532-5415.1996.tb03730.x 8675921

[B10] Brentano M. A. Cadore E. L. Da Silva E. M. Ambrosini A. B. Coertjens M. Petkowicz R. (2008). Physiological adaptations to strength and circuit training in postmenopausal women with bone loss. J. Strength Cond. Res. 22, 1816–1825. 10.1519/JSC.0b013e31817ae3f1 18978624

[B11] Burge R. Dawson-Hughes B. Solomon D. H. Wong J. B. King A. Tosteson A. (2007). Incidence and economic burden of osteoporosis-related fractures in the United States, 2005-2025. J. Bone Min. Res. 22, 465–475. 10.1359/jbmr.061113 17144789

[B12] Chahal J. Lee R. Luo J. (2014). Loading dose of physical activity is related to muscle strength and bone density in middle-aged women. Bone 67, 41–45. 10.1016/j.bone.2014.06.029 24999224

[B13] Cheng J. Meng S. Lee J. Kwak H.-B. Liu Y. (2022). Effects of walking and sun exposure on bone density and balance in elderly with osteopenia. J. Bone Min. Metab. 40, 528–534. 10.1007/s00774-022-01317-7 35347429

[B14] Cheung A. M. Giangregorio L. (2012). Mechanical stimuli and bone health. Curr. Opin. Rheumatol. 24, 561–566. 10.1097/BOR.0b013e3283570238 22832826

[B65] Chien M. Y. Wu Y. T. Hsu A. T. Yang R. S. Lai J. S. (2000). Efficacy of a 24-week aerobic exercise program for osteopenic postmenopausal women. Calcif. Tissue Int. 67 (6), 443–448. 10.1007/s002230001180 11289692

[B15] Consensus development conference: Diagnosis, prophylaxis, and treatment of osteoporosis (1993). Consensus development conference: Diagnosis, prophylaxis, and treatment of osteoporosis. Am. J. Med. 94, 646–650. 10.1016/0002-9343(93)90218-e 8506892

[B16] Cumpston M. Li T. Page M. J. Chandler J. Welch V. A. Higgins J. P. (2019). Updated guidance for trusted systematic reviews: A new edition of the Cochrane Handbook for systematic reviews of interventions. Cochrane Database Syst. Rev. 10, ED000142. 10.1002/14651858.ED000142 31643080 PMC10284251

[B17] Deeks J. J. Higgins J. P. Altman D. G. Group, on behalf of the C. S. M (2019). “Analysing data and undertaking meta-analyses,” in Cochrane Handbook for systematic reviews of interventions (John Wiley & Sons, Ltd). 241–284. 10.1002/9781119536604.ch10

[B18] ElDeeb A. M. Abdel-Aziem A. A. (2020). Effect of whole-body vibration exercise on power profile and bone mineral density in postmenopausal women with osteoporosis: A randomized controlled trial. J. Manip. Physiol. Ther. 43, 384–393. 10.1016/j.jmpt.2019.12.003 32868028

[B19] Ernst E. (1998). Exercise for female osteoporosis - a systematic review of randomised clinical trials. Sports Med. 25, 359–368. 10.2165/00007256-199825060-00002 9680658

[B20] Filipović T. N. Lazović M. P. Backović A. N. Filipović A. N. Ignjatović A. M. Dimitrijević S. S. (2021). A 12-week exercise program improves functional status in postmenopausal osteoporotic women: Randomized controlled study. Eur. J. Phys. Rehabil. Med. 57, 120–130. 10.23736/S1973-9087.20.06149-3 32902207

[B21] Garber C. E. Blissmer B. Deschenes M. R. Franklin B. A. Lamonte M. J. Lee I.-M. (2011). American College of Sports medicine position stand. Quantity and quality of exercise for developing and maintaining cardiorespiratory, musculoskeletal, and neuromotor fitness in apparently healthy adults: Guidance for prescribing exercise. Med. Sci. Sports Amp Exerc. 43, 1334–1359. 10.1249/MSS.0b013e318213fefb 21694556

[B22] Gregson C. L. Armstrong D. J. Bowden J. Cooper C. Edwards J. Gittoes N. J. L. (2022). UK clinical guideline for the prevention and treatment of osteoporosis. Arch. Osteoporos. 17, 58. 10.1007/s11657-022-01061-5 35378630 PMC8979902

[B23] Hadji P. Klein S. Gothe H. Häussler B. Kless T. Schmidt T. (2013). The epidemiology of osteoporosis-bone evaluation study (BEST): An analysis of routine health insurance data. Dtsch. Ärztebl. Int. 110, 52–57. 10.3238/arztebl.2013.0052 23413388 PMC3570954

[B24] Hakestad K. A. Torstveit M. K. Nordsletten L. Risberg M. A. (2015). Effect of exercises with weight vests and a patient education programme for women with osteopenia and a healed wrist fracture: A randomized, controlled trial of the OsteoACTIVE programme. Bmc Musculoskelet. Disord. 16, 352. 10.1186/s12891-015-0811-z 26578370 PMC4650105

[B64] Hartard M. Haber P. Ilieva D. Preisinger E. Seidl G. Huber J. (1996). Systematic strength training as a model of therapeutic intervention. A controlled trial in postmenopausal women with osteopenia. Am. J. Phys. Med. Rehabil. 75 (1), 21–28. 10.1097/00002060-199601000-00006 8645434

[B25] Hettchen M. von Stengel S. Kohl M. Murphy M. H. Shojaa M. Ghasemikaram M. (2021). Changes in menopausal risk factors in early postmenopausal osteopenic women after 13 Months of high-intensity exercise: The randomized controlled ACTLIFE-RCT. Clin. Interv. Aging 16, 83–96. 10.2147/CIA.S283177 33469276 PMC7810823

[B26] Higgins J. P. T. Altman D. G. Gotzsche P. C. Juni P. Moher D. Oxman A. D. (2011). The Cochrane Collaboration’s tool for assessing risk of bias in randomised trials. BMJ 343, d5928. 10.1136/bmj.d5928 22008217 PMC3196245

[B66] Hourigan S. R. Nitz J. C. Brauer S. G. O'Neill S. Wong J. Richardson C. A. (2008). Positive effects of exercise on falls and fracture risk in osteopenic women. Osteoporos Int. 19, 1077–1086. 10.1007/s00198-007-0541-7 18188658

[B27] Iwamoto J. Takeda T. Ichimura S. (2001). Effect of exercise training and detraining on bone mineral density in postmenopausal women with osteoporosis. J. Orthop. Sci. 6, 128–132. 10.1007/s007760100059 11484097

[B28] Iwamoto J. Takeda T. Otani T. Yabe Y. (1998). Effect of increased physical activity on bone mineral density in postmenopausal osteoporotic women. Keio J. Med. 47, 157–161. 10.2302/kjm.47.157 9785761

[B29] Kelley G. A. Kelley K. S. Tran Z. V. (2001). Resistance training and bone mineral density in women: A meta-analysis of controlled trials. Am. J. Phys. Med. Rehabil. 80, 65–77. 10.1097/00002060-200101000-00017 11138958

[B30] Kemmler W. Shojaa M. Kohl M. von Stengel S. (2020). Effects of different types of exercise on bone mineral density in postmenopausal women: A systematic review and meta-analysis. Calcif. Tissue Int. 107, 409–439. 10.1007/s00223-020-00744-w 32785775 PMC7546993

[B31] Kemmler W. von Stengel S. (2014). Dose-response effect of exercise frequency on bone mineral density in post-menopausal, osteopenic women. Scand. J. Med. Sci. Sports 24, 526–534. 10.1111/sms.12024 23190199

[B32] Kienberger Y. Sassmann R. Rieder F. Johansson T. Kassmann H. Pirich C. (2022). Effects of whole body vibration in postmenopausal osteopenic women on bone mineral density, muscle strength, postural control and quality of life: The T-bone randomized trial. Eur. J. Appl. Physiol. 122, 2331–2342. 10.1007/s00421-022-05010-5 35864343 PMC9560973

[B33] Kistler-Fischbacher M. Yong J. S. Weeks B. K. Beck B. R. (2021). A comparison of bone-targeted exercise with and without antiresorptive bone medication to reduce indices of fracture risk in postmenopausal women with low bone mass: The MEDEX-OP randomized controlled trial. J. Bone Min. Res. 36, 1680–1693. 10.1002/jbmr.4334 34033146

[B34] Kitagawa T. Hiraya K. Denda T. Yamamoto S. (2022). A comparison of different exercise intensities for improving bone mineral density in postmenopausal women with osteoporosis: A systematic review and meta-analysis. Bone Rep. 17, 101631. 10.1016/j.bonr.2022.101631 36310762 PMC9615132

[B35] Korpelainen R. Keinanen-Kiukaanniemi S. Heikkinen J. Vaananen K. Korpelainen J. (2006). Effect of impact exercise on bone mineral density in elderly women with low BMD: A population-based randomized controlled 30-month intervention. Osteoporos. Int. 17, 109–118. 10.1007/s00198-005-1924-2 15889312

[B36] LeBoff M. S. Greenspan S. L. Insogna K. L. Lewiecki E. M. Saag K. G. Singer A. J. (2022). The clinician’s guide to prevention and treatment of osteoporosis. Osteoporos. Int. 33, 2049–2102. 10.1007/s00198-021-05900-y 35478046 PMC9546973

[B37] Lin Y. A. Chen L. H. Chen F. P. Wong A.-K. Hsu C. C. Chen J. Y. (2022). The effectiveness of a group kickboxing training program on sarcopenia and osteoporosis parameters in community-dwelling adults aged 50–85 years. Front. Med. 9, 815342. 10.3389/fmed.2022.815342 PMC9081979 35547204

[B38] Liu B. X. Chen S. P. Li Y. D. Wang J. Zhang B. Lin Y. (2015). The effect of the modified eighth section of eight-section brocade on osteoporosis in postmenopausal women A prospective randomized trial. Med. Baltim. 94, e991. 10.1097/md.0000000000000991 PMC4504582 26107684

[B39] Liu F. H. Wang S. (2017). Effect of Tai Chi on bone mineral density in postmenopausal women: A systematic review and meta-analysis of randomized control trials. J. Chin. Med. Assoc. 80, 790–795. 10.1016/j.jcma.2016.06.010 28827032

[B40] Liu-Ambrose T. Y. Khan K. M. Eng J. J. Heinonen A. McKay H. A. (2004). Both resistance and agility training increase cortical bone density in 75- to 85-year-old women with low bone mass: A 6-month randomized controlled trial. J. Clin. Densitom. 7, 390–398. 10.1385/jcd:7:4:390 15618599

[B67] Marchese D. D'andrea M. Ventura V. Montalcini T. Foti D. Pujia A. (2012). Effects of a weight-bearing exercise training on bone mineral density and neuromuscular function of osteopenic women. Eur. J. Inflamm. 10 (3), 427–435. 10.1177/1721727X1201000318

[B41] Marcu R. I. Patru S. Bighea A. C. Traistaru R. Popescu R. S. (2015). FRI0288 Role of physical exercice program in patients with osteoporosis. Ann. Rheum. Dis. 74, 521–529. 10.1136/annrheumdis-2015-eular.5030

[B42] Moseng T. Dagfinrud H. Smedslund G. Østerås N. (2017). The importance of dose in land-based supervised exercise for people with hip osteoarthritis. A systematic review and meta-analysis. Osteoarthr. Cartil. 25, 1563–1576. 10.1016/j.joca.2017.06.004 28648741

[B68] Mosti M. P. Kaehler N. Stunes A. K. Hoff J. Hoff J. Syversen U. (2013). Maximal strength training in postmenopausal women with osteoporosis or osteopenia. J. Strength Cond. Res. 27 (10), 2879–2886. 10.1519/JSC.0b013e318280d4e2 23287836

[B43] Mu W. Huang X. Zhang J. Liu X. Huang M. (2018). Effect of Tai Chi for the prevention or treatment of osteoporosis in elderly adults: Protocol for a systematic review and meta-analysis. BMJ Open 8, e020123. 10.1136/bmjopen-2017-020123 PMC5892770 29632082

[B44] Nelson M. E. Fiatarone M. A. Morganti C. M. Trice I. Greenberg R. A. Evans W. J. (1994). Effects of high-intensity strength training on multiple risk-factors for osteoporotic fractures - a randomized controlled trial. Jama-J. Am. Med. Assoc. 272, 1909–1914. 10.1001/jama.1994.03520240037038 7990242

[B45] Pedersen B. K. Saltin B. (2015). Exercise as medicine - evidence for prescribing exercise as therapy in 26 different chronic diseases. Scand. J. Med. Sci. Sports 25, 1–72. 10.1111/sms.12581 26606383

[B46] Posch M. Schranz A. Lener M. Tecklenburg K. Burtscher M. Ruedl G. (2019). Effectiveness of a mini-trampoline training program on balance and functional mobility, gait performance, strength, fear of falling and bone mineral density in older women with osteopenia. Clin. Interv. Aging 14, 2281–2293. 10.2147/cia.S230008 31908438 PMC6929928

[B47] Rajapakse C. S. Johncola A. J. Batzdorf A. S. Jones B. C. Al Mukaddam M. Sexton K. (2021). Effect of low-intensity vibration on bone strength, microstructure, and adiposity in pre-osteoporotic postmenopausal women: A randomized placebo-controlled trial. J. Bone Min. Res. 36, 673–684. 10.1002/jbmr.4229 33314313

[B48] Shen Z. F. Zhu G. F. Qian L. F. Fu Y. X. (2018). Yi Jin jing (Sinew-transforming qigong exercises) for primary osteoporosis in the elderly: A clinical trial. J. Acupunct. Tuina Sci. 16, 104–108. 10.1007/s11726-018-1032-4

[B49] Shojaa M. von Stengel S. Kohl M. Schoene D. Kemmler W. (2020). Effects of dynamic resistance exercise on bone mineral density in postmenopausal women: A systematic review and meta-analysis with special emphasis on exercise parameters. Osteoporos. Int. 31, 1427–1444. 10.1007/s00198-020-05441-w 32399891 PMC7360540

[B50] Skjødt M. K. Frost M. Abrahamsen B. (2019). Side effects of drugs for osteoporosis and metastatic bone disease. Br. J. Clin. Pharmacol. 85, 1063–1071. 10.1111/bcp.13759 30192026 PMC6533454

[B51] Sterne J. A. C. Savović J. Page M. J. Elbers R. G. Blencowe N. S. Boutron I. (2019). RoB 2: A revised tool for assessing risk of bias in randomised trials. BMJ 366, l4898. 10.1136/bmj.l4898 31462531

[B52] Sugerman D. T. (2014). JAMA patient page. Osteoporosis. JAMA 311, 104. 10.1001/jama.2013.283009 24381978

[B53] Sun C. Qi B. Huang X. Chen M. Jin Z. Zhang Y. (2022). Baduanjin exercise: A potential promising therapy toward osteoporosis. Front. Med. Lausanne 9, 935961. 10.3389/fmed.2022.935961 35991646 PMC9381703

[B54] Svedbom A. Hernlund E. Ivergård M. Compston J. Cooper C. Stenmark J. (2013). Osteoporosis in the European union: A compendium of country-specific reports. Arch. Osteoporos. 8, 137. 10.1007/s11657-013-0137-0 24113838 PMC3880492

[B55] Tong X. Chen X. Zhang S. Huang M. Shen X. Xu J. (2019). The effect of exercise on the prevention of osteoporosis and bone angiogenesis. Biomed. Res. Int. 2019, 8171897. 10.1155/2019/8171897 31139653 PMC6500645

[B56] Waltman N. Kupzyk K. A. Flores L. E. Mack L. R. Lappe J. M. Bilek L. D. (2022). Bone-loading exercises versus risedronate for the prevention of osteoporosis in postmenopausal women with low bone mass: A randomized controlled trial. Osteoporos. Int. 33, 475–486. 10.1007/s00198-021-06083-2 34519832

[B69] Wang H. Yu B. Chen W. Lu Y. Yu D. (2015). Simplified Tai Chi resistance training versus traditional Tai Chi in slowing bone loss in postmenopausal women. Evid. Based Complement Alternat. Med. 2015, 379451. 10.1155/2015/379451 26136808 PMC4475529

[B57] Watson S. L. Weeks B. K. Weis L. J. Harding A. T. Horan S. A. Beck B. R. (2018). High-intensity resistance and impact training improves bone mineral density and physical function in postmenopausal women with osteopenia and osteoporosis: The LIFTMOR randomized controlled trial. J. Bone Min. Res. 33, 211–220. 10.1002/jbmr.3284 28975661

[B58] Watson S. L. Weeks B. K. Weis L. J. Horan S. A. Beck B. R. (2015). Heavy resistance training is safe and improves bone, function, and stature in postmenopausal women with low to very low bone mass: Novel early findings from the LIFTMOR trial. Osteoporos. Int. 26, 2889–2894. 10.1007/s00198-015-3263-2 26243363

[B59] Wayne P. M. Kiel D. P. Buring J. E. Connors E. M. Bonato P. Yeh G. Y. (2012). Impact of Tai Chi exercise on multiple fracture-related risk factors in post-menopausal osteopenic women: A pilot pragmatic, randomized trial. Bmc Complement. Altern. Med. 12, 7. 10.1186/1472-6882-12-7 22289280 PMC3298524

[B60] Wei X. Xu A. L. Yin Y. K. Zhang R. X. (2015). The potential effect of wuqinxi exercise for primary osteoporosis: A systematic review and meta-analysis. Maturitas 82, 346–354. 10.1016/j.maturitas.2015.08.013 26386831

[B61] Yu P. A. Hsu W. H. Hsu W. B. Kuo L. T. Lin Z. R. Shen W. J. (2019). The effects of high impact exercise intervention on bone mineral density, physical fitness, and quality of life in postmenopausal women with osteopenia: A retrospective cohort study. Med. Baltim. 98, e14898. 10.1097/md.0000000000014898 PMC6426501 30882707

[B62] Zeng Q. Li N. Wang Q. Feng J. Sun D. Zhang Q. (2019). The prevalence of osteoporosis in China, a nationwide, multicenter DXA survey. J. Bone Min. Res. 34, 1789–1797. 10.1002/jbmr.3757 31067339

[B63] Zhang S. Huang X. Zhao X. Li B. Cai Y. Liang X. (2022). Effect of exercise on bone mineral density among patients with osteoporosis and osteopenia: A systematic review and network meta‐analysis. J. Clin. Nurs. 31, 2100–2111. 10.1111/jocn.16101 34725872

