# Supplementary material for: Effects of exercise based on ACSM recommendations on bone mineral density in individuals with osteoporosis: a systematic review and meta-analyses of randomized controlled trials
Source: Front Physiol. 2023 Jul 17;14:1181327. doi: 10.3389/fphys.2023.1181327 (PMC10389279; doi:10.3389/fphys.2023.1181327)

Supplementary Material

Effects of exercise based on ACSM recommendations on bone mineral density in individuals with osteoporosis: A systematic review and meta-analyses of randomized controlled trials

Wenlai Cui^1^, Dong Li^2^, Yueshuai Jiang^1^, Yang Gao^1*^

*** Correspondence:** Yang Gao: gaoyang@cupes.edu.cn

**Appendix 1** Search Strategy

| Database | Search strategy | amount |
| --- | --- | --- |
|  | PubMed |  |
| #1 | Search: "Osteoporosis"[Mesh] Sort by: Most Recent | 61733 |
| #2 | Search: (((((((((((Osteoporosis[Title/Abstract]) OR (Osteoporoses[Title/Abstract])) OR (osteopenic[Title/Abstract])) OR (osteoporotic[Title/Abstract])) OR (osteopenia[Title/Abstract])) OR (Post-Traumatic Osteoporoses[Title/Abstract])) OR (Senile Osteoporoses[Title/Abstract])) OR (Bone Loss[Title/Abstract])) OR (Bone Losses[Title/Abstract])) OR (Bone density[Title/Abstract])) OR (Bone mineral density[Title/Abstract])) OR (Low bone mass[Title/Abstract]) | 147746 |
| #3 | #1 OR #2 | 157853 |
| #4 | Search: "Exercise"[Mesh] Sort by: Most Recent | 239765 |
| #5 | Search: (((((((((((((((Exercises[Title/Abstract]) OR (Physical Activity[Title/Abstract])) OR (physical exercise[Title/Abstract])) OR (Training[Title/Abstract])) OR (Trainings[Title/Abstract])) OR (Motor Activity[Title/Abstract])) OR (Tai Chi[Title/Abstract])) OR (Vibration[Title/Abstract])) OR (yoga[Title/Abstract])) OR (wuqinxi[Title/Abstract])) OR (baduanjin[Title/Abstract])) OR (yijinjing[Title/Abstract])) OR (kickboxing[Title/Abstract])) OR (Pilates[Title/Abstract])) OR (Balance[Title/Abstract])) OR (Resistance[Title/Abstract]) | 1835783 |
| #6 | #4 OR #5 | 1954224 |
| #7 | Search: ((((Randomized controlled trial[Publication Type]) OR (controlled clinical trial[Publication Type])) OR (randomized[Title/Abstract])) OR (placebo[Title/Abstract])) OR (randomly[Title/Abstract]) | 1296421 |
| #8 | #3 AND #6 AND #7 | 2075 |
|  | Embase |  |
| #1 | 'osteoporosis'/exp OR osteoporosis OR osteoporoses:ab,ti OR osteopenic:ab,ti OR osteoporotic:ab,ti OR osteopenia:ab,ti OR 'post-traumatic osteoporoses':ab,ti OR 'senile osteoporoses':ab,ti OR 'bone loss':ab,ti OR 'bone losses':ab,ti OR 'bone density':ab,ti OR 'bone mineral density':ab,ti OR 'low bone mass':ab,ti | 262102 |
| #2 | ('exercise'/exp OR exercise OR exercises:ab,ti OR 'physical activity':ab,ti OR 'physical exercise':ab,ti OR training:ab,ti OR trainings:ab,ti OR 'motor activity':ab,ti OR 'tai chi':ab,ti OR vibration:ab,ti OR yoga:ab,ti OR wuqinxi:ab,ti OR baduanjin:ab,ti OR yijinjing:ab,ti OR kickboxing:ab,ti OR pilates:ab,ti OR balance:ab,ti OR resistance:ab,ti) AND ([controlled clinical trial]/lim OR [randomized controlled trial]/lim) | 149795 |
| #3 | #1 AND #2 | 2324 |
|  | Web of Science |  |
| #1 | ((((((((((((ALL=(Osteoporosis)) OR TS=(Osteoporosis )) OR TS=(Osteoporoses )) OR TS=(osteopenic )) OR TS=(osteoporotic )) OR TS=(osteopenia )) OR TS=( Post-Traumatic Osteoporoses )) OR TS=(Senile Osteoporoses )) OR TS=(Bone Loss )) OR TS=(Bone Losses )) OR TS=(Bone density )) OR TS=(Bone mineral density )) OR TS=(Low bone mass) | 283073 |
| #2 | ((((((((((((((((ALL=(Exercise)) OR TS=(Exercises)) OR TS=(Physical Activity )) OR TS=(physical exercise)) OR TS=( Training )) OR TS=(Trainings)) OR TS=( Motor Activity)) OR TS=( Tai Chi )) OR TS=(Vibration )) OR TS=(yoga )) OR TS=( wuqinxi )) OR TS=( baduanjin )) OR TS=(yijinjing )) OR TS=(kickboxing )) OR TS=(Pilates)) OR TS=( Balance)) OR TS=( Resistance) | 5167580 |
| #3 | ((((TS=(Randomized controlled trial )) OR TS=(controlled clinical trial )) OR TS=( randomized )) OR TS=(placebo )) OR TS=( randomly) | 1584625 |
| #4 | #1 AND #2 AND #3 | 4827 |
|  | Cochrane |  |
| #1 | (Osteoporosis):ti,ab,kw OR (Osteoporoses):ti,ab,kw OR (osteopenic):ti,ab,kw OR (osteoporotic):ti,ab,kw OR (osteopenia):ti,ab,kw | 12942 |
| #2 | (Post-Traumatic Osteoporoses):ti,ab,kw OR (Senile Osteoporoses):ti,ab,kw OR (Bone Loss):ti,ab,kw OR (Bone Losses):ti,ab,kw OR (Bone density):ti,ab,kw | 20858 |
| #3 | (Bone mineral density):ti,ab,kw OR (Low bone mass):ti,ab,kw | 10249 |
| #4 | #1 OR #2 OR #3 | 26856 |
| #5 | (Exercise):ti,ab,kw OR (Exercises):ti,ab,kw OR (Physical Activity):ti,ab,kw OR (physical exercise):ti,ab,kw OR (Training):ti,ab,kw | 245500 |
| #6 | (Trainings):ti,ab,kw OR (Motor Activity):ti,ab,kw OR (Tai Chi):ti,ab,kw OR (Vibration):ti,ab,kw OR (yoga):ti,ab,kw | 153675 |
| #7 | (wuqinxi):ti,ab,kw OR (baduanjin):ti,ab,kw OR (yijinjing):ti,ab,kw OR (kickboxing):ti,ab,kw OR (Pilates):ti,ab,kw | 1273 |
| #8 | (Balance):ti,ab,kw OR (Resistance):ti,ab,kw | 131259 |
| #9 | #5 OR #6 OR #7 OR #8 | 350800 |
| #10 | (Randomized controlled trial):ti,ab,kw OR (controlled clinical trial):ti,ab,kw OR (randomized):ti,ab,kw | 1257405 |
| #11 | #4 AND #9 AND #10 | 4236 |

**Appendix 2** Funnel plot of lumbar spine BMD in resistance exercise


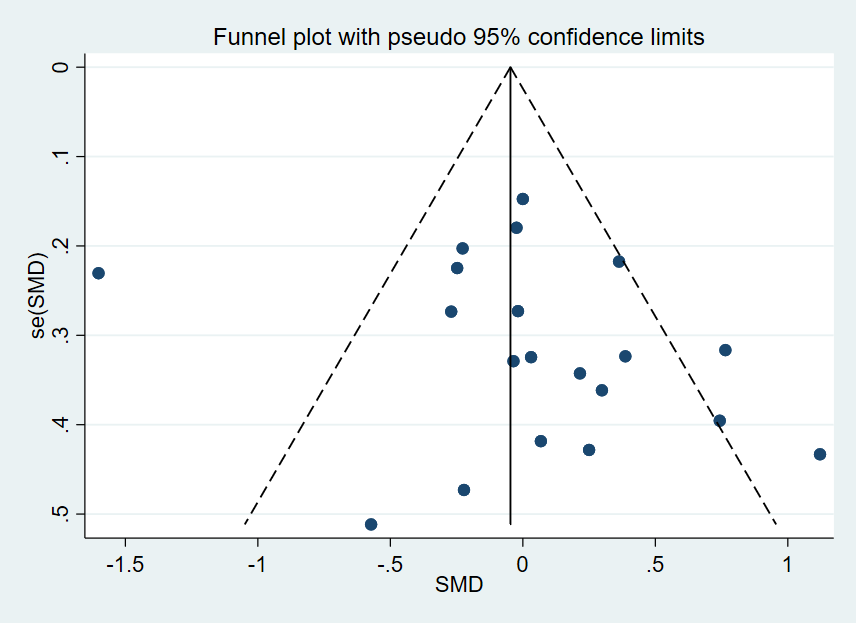


**Appendix 3** Sensitivity analyses of lumbar spine BMD in resistance exercise


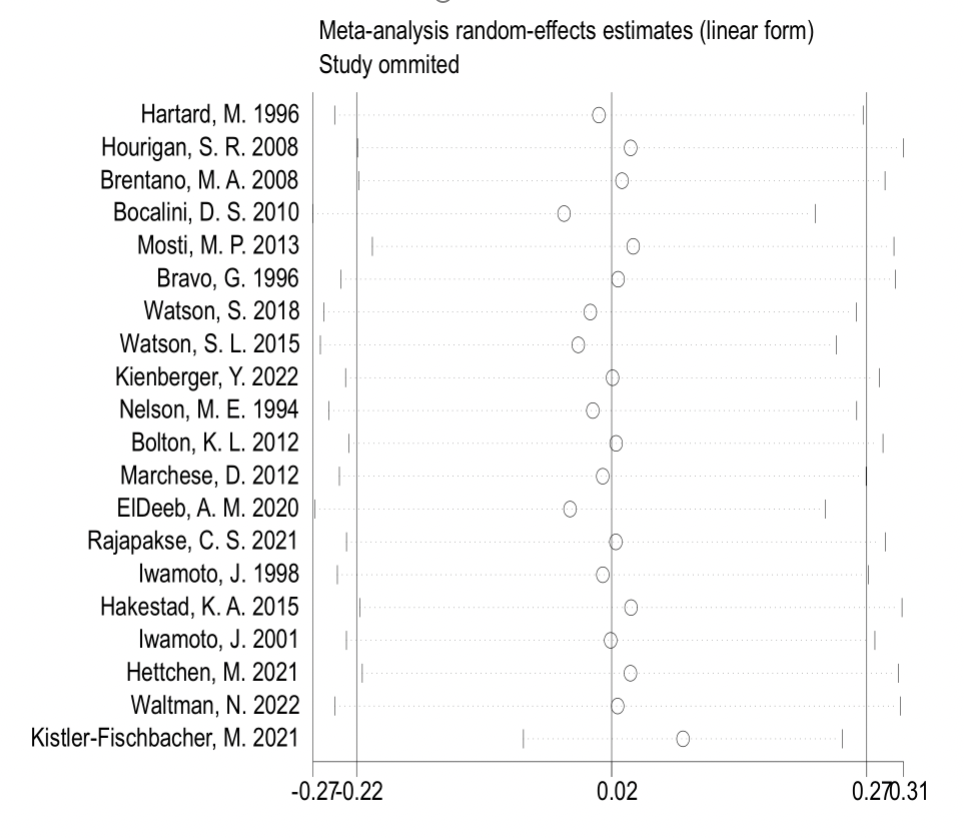


**Appendix 4** Funnel plot of femoral neck BMD in resistance exercise


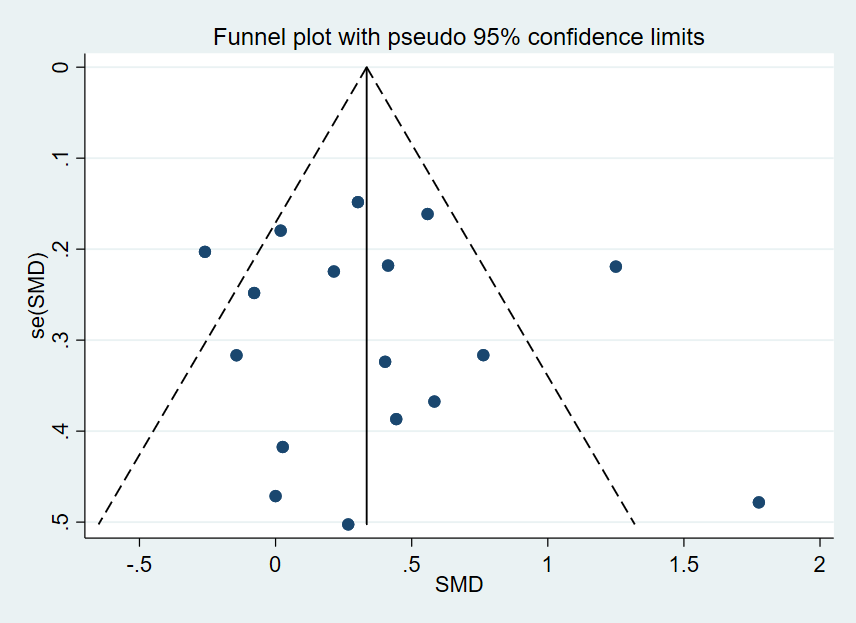


**Appendix 5** Sensitivity analyses of femoral neck BMD in resistance exercise


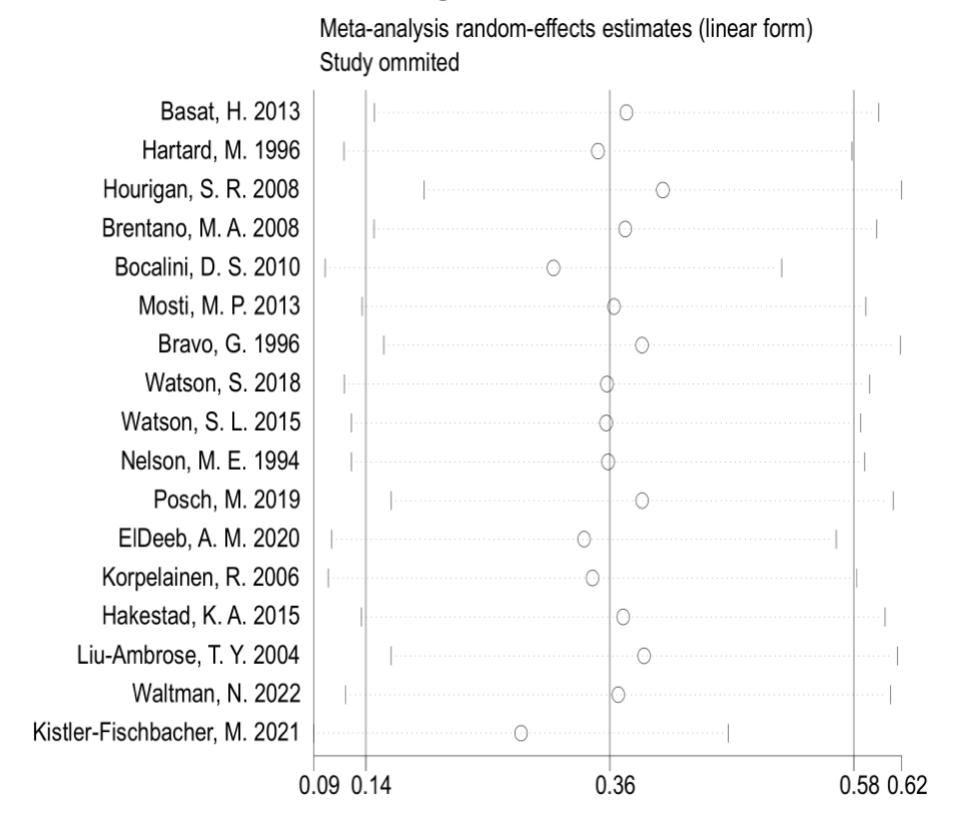

Supplement: Supplementary file 1 [file DataSheet1.docx]
